# Supplementary material for: Connectivity in Spanish metapopulation of Dupont’s lark may be maintained by dispersal over medium-distance range and stepping stones
Source: PeerJ. 2021 Aug 19;9:e11925. doi: 10.7717/peerj.11925 (PMC8380426; doi:10.7717/peerj.11925)
Supplement: Supplemental Information 1 — Year and month (season) of observation, locality and province, nearest subpopulation name and province (see Supplemental Table S3 for a complete list of names), and minimum distance to the nearest current subpopulation are provided, as well the reference where the data are provided, and other useful information. [file peerj-09-11925-s001.docx]

| Year | Month/season | Locality | Prov. | Nearest subp. | Prov. | Dist. | Reference | Other available information |
| --- | --- | --- | --- | --- | --- | --- | --- | --- |
| pre-2000 | *Unknown* | Málaga | Málaga | Padul | Granada | 70.56 | Irby, 1895 |  |
| 1874 | December | Real Sitio de San Ildefonso | Segovia | Hoces del Duratón | Segovia | 45.10 | Castellarnau, 1877 | Two observations, one of the next to the road to Segovia |
| 1889 | *Unknown* | Mataró | Barcelona | Alfés | Lérida | 145.82 | Cordero, in Muntaner *et al.*, 1984 |  |
| 1896 | *Unknown* | Cànoes | Rosselló (France) | Alfés | Lérida | 215.18 | Ferrer *et al.*, 1986 |  |
| 1923 | February | Gavá | Barcelona | Alfés | Lérida | 112.51 | Segarra, in Gutiérrez *et al.*, 1995 | 1 individual captured together with Eurasian Skylarks (*Alauda arvensis*) |
| 1925 | *Unknown* | Casteldefells | Barcelona | Alfés | Lérida | 111.78 | Mestre Raventós, 1969 | 1 individual collected, Museum of Zoology of Barcelona |
| 1960's | Autumn | Alcazar de San Juan | Ciudad Real | Pastrana (*) | Toledo | 9.12 | Bernis, 1971 |  |
| 1960's | *Unknown* | Palma de Mallorca | Mallorca | Alfés | Lérida | 274.13 | Suárez and Garza, 1989 | In Madrid Natural Science Museum |
| 1960's | Autumn | Torrecaballeros | Segovia | Hoces del Duratón | Segovia | 35.93 | Bernis, 1971 |  |
| 1963 | February | Torredembarra | Tarragona | Alfés | Lérida | 74.13 | Mestre Raventós, 1969 | Several observations in the beach and sand dunes, 4 individuals captured (1 male, 2 females, 1 unknown) |
| 1970's | *Unknown* | Alicante | Alicante | Herrada del Manco | Murcia | 54.00 | Bernis; in de Juana, 1980 |  |
| 1970's | *Unknown* | Mizala | Almería | Karst de Sorbas | Almería | 5.23 | Bernis; in Manrique, 1993 |  |
| 1977 | Spring | Lozoyuela | Madrid | Hoces del Duratón | Segovia | 41.86 | Tellería, J. L., pers. com. | 1 individual |
| 1978 | August 5^th^ | Guadix | Granada | Cerro Villegas (*) | Almería | 33.53 | de Juana, 1983 | Flock of 20 individuals foraging on the ground between Guadix and Baza |
| 1980's | May | Gea y Truyols | Murcia | Llano de las Cabras (*) | Murcia | 52.12 | Brotons, 1989 | 1 individual |
| 1980's | Winter | Guadalete River, drain marsh | Cádiz | Padul | Granada | 173.78 | Ceballos and Guimerá, 1992 | 1 observation |
| 1980's | *Unknown* | Venta Seca | Murcia | Llano de las Cabras (*) | Murcia | 23.63 | López *et al.;* in Guardiola *et al.*, 2000 |  |
| *Unknown* | *Unknown* | Cariñena | Zaragoza | Longares-Mezalocha | Zaragoza | 8.24 | Navas; in de Juana, 1980 |  |
| 1981 | *Unknown* | Villalta | Burgos | Páramo de Castañeda (*) | Palencia | 110.84 | Suárez *et al.*, 1982 |  |
| 1982 | *Unknown* | Pedro-Martínez | Granada | Cerro Villegas (*) | Almería | 49.83 | Pleguezuelos, 1992 |  |
| 1985 | Spring | El Pedroso de la Armuña | Salamanca | Almaraz de Duero | Zamora | 54.83 | Peris and Carnero, 1988 | 1 individual observed |
| 1986 | May | Alcanadre | La Rioja | Balsa del Pulguer (*) | Navarra | 48.17 | Domingo and Nalda, 1987 | 1 individual |
| 1987 | June 16^th^ | Punta Umbría, Odiel Marsh | Huelva | Padul | Granada | 288.67 | Jiménez Vázquez, 1987 | 1 adult male captured with mist net |
| 1988-1989 | Breeding season | Trebujena-Sanlucar, drain marsh | Cádiz | Padul | Granada | 224.77 | Ceballos and Guimerá, 1992 | Presence of individuals |
| 1990 | *Unknown* | Darro | Granada | Padul | Granada | 47.20 | Hodar; in Suárez, 2010 |  |
| 1990's | *Unknown* | Cabañeros | Ciudad Real | Pastrana (*) | Toledo | 81.02 | J. Jiménez, pers. com | 1 individual found dead |
| 1994 | Spring | Cardenete | Cuenca | Cardenete | Cuenca | 3.47 | Garza; in Suárez, 2010 | 1 male calling |
| 1996 | March | Fuente la Mora | Valladolid | Páramo de Castañeda (*) | Palencia | 29.73 | Aguado, 1996 | 1 individual |
| 1998 | February | Granja de San Andrés de Valvení | Valladolid | Páramo de Castañeda (*) | Palencia | 13.83 | Decimavilla, 1998 | 1 individual |
| 1998 | May | Santo Domingo de Pirón | Segovia | Hoces del Duratón | Segovia | 30.19 | Casaux; in Suárez, 2010 |  |
| 1999 | May | Cantalapiedra | Salamanca | Almaraz de Duero | Zamora | 65.50 | Pescador, 1999 | 1 individual |
| 1999 | July | Golpejas-Vega de Tirados | Salamanca | Almaraz de Duero | Zamora | 45.13 | López; in Sanz-Zuasti and García Fernández, 2006 | 1 male singing |
| 1999 | July | San Martín de Valvení | Valladolid | Páramo de Castañeda (*) | Palencia | 15.68 | Decimavilla, 1999 | 1 individual |
| 1999 | July | Villena | Alicante | Herrada del Manco | Murcia | 13.06 | Cabo *et al.*, 2003 | 1 individual |
| 2000 | *Unknown* | Aldeadávila de la Ribera | Salamanca | Fariza | Zamora | 31.81 | López and Cuadrado; in Sanz-Zuasti and García Fernández, 2006 | 1 individual observed |
| 2000 | *Unknown* | El Hito lagoon | Cuenca | Zafra de Záncara | Cuenca | 9.62 | Velasco; in Velasco *et al*., 2001 | 1 individual |
| 2000 | *Unknown* | Mieza | Salamanca | Fariza | Zamora | 40.45 | López and Cuadrado; in Sanz-Zuasti and García Fernández, 2006 | 1 individual observed |
| 2000, aprox. | *Unknown* | Belinchón | Cuenca | Saelices | Cuenca | 12.66 | Velasco *et al*., 2001 | 1 observation |
| 2000, aprox. | *Unknown* | Cartagena, Marina de Carmol | Murcia | Llano de las Cabras (*) | Murcia | 60.10 | Blanco; in Guardiola *et al.*, 2000 |  |
| 2002, aprox. | *Unknown* | Guadahortuna | Granada | Padul | Granada | 61.06 | Pérez; in Suárez, 2010 |  |
| 2001 | Winter | Tembleque | Toledo | Lillo (*) | Toledo | 14.05 | T. Gullick, pers. com. | 1 individual, in old stubble |
| *Unknown* | *Unknown* | Belinchón | Cuenca | Saelices | Cuenca | 12.63 | Velasco; in Velasco *et al*., 2001 |  |
| *Unknown* | *Unknown* | Pozo Cañada | Albacete | Hoya Gonzalo | Albacete | 19.55 | Campos *et al.*, 2001 |  |
| 2002 | *Unknown* | Abarán, LLanos de Casablanca | Murcia | Sierra del Picarcho | Murcia | 11.54 | Barba and Gómez, 2002 |  |
| 2002 | April | Brazo del Este | Sevilla | Padul | Granada | 206.46 | García Vargas *et al.*, 2003 | 1 individual |
| 2002 | October 27^th^ | Pozo Sastre, LLano de Orce | Granada | Cerro Villegas (*) | Almería | 54.93 | Palanca, 2004 | 1 male singing during 15 minutes |
| 2002 | July | Pozorrubio de Santiago | Cuenca | Saelices | Cuenca | 15.65 | T. Gullick, pers. com. | 1 individual, in recent stubble, Torrelaguna estate |
| 2002 | January | Vega de Carmona | Sevilla | Padul | Granada | 176.65 | García Vargas, 2002 | 1 individual |
| 2003 | May | Serradiel, Casas Ibáñez | Albacete | Hoya Gonzalo | Albacete | 25.70 | Picazo y Miñano, 2003 | 1 individual |
| 2003 | June | Totana, Collado Bermejo | Murcia | Llano de las Cabras (*) | Murcia | 9.91 | Hernández, 2003 | 3 individuals |
| 2005 | *Unknown* | Darro | Granada | Padul | Granada | 47.20 | Garzón; in Suárez, 2010 |  |
| 2010 | January 9^th^ | Albufera Natural Park | Valencia | Herrada del Manco | Murcia | 98.77 | Dies *et al.*, 2010 | 1 individual, observed until January 12^th^ and never again, period of snow storms in the interior highlands |
| 2015 | Summer | Alcázar de San Juan | Ciudad Real | Pastrana (*) | Toledo | 9.12 | P. Bustamante, pers. com. | 1 individual photographied |
| 2015 | March 28^th^ | La Manga | Murcia | Llano de las Cabras (*) | Murcia | 80.40 | García and Requena, 2015 | 1 individual in a Short-toed Lark (*Calandrella brachydactyla*) flock, no presence in following days |
| 2019 | September | Campaspero | Valladolid | Páramo de Corcos | Burgos | 32.52 | A. Fernández Ortiz, pers. com. |  |
