# Supplementary material for: Connectivity in Spanish metapopulation of Dupont’s lark may be maintained by dispersal over medium-distance range and stepping stones
Source: PeerJ. 2021 Aug 19;9:e11925. doi: 10.7717/peerj.11925 (PMC8380426; doi:10.7717/peerj.11925)
Supplement: Supplemental Information 2 — Codes and description of CORINE land use categories that accounted for 95% of Dupont’s Lark post-2000 observations, and were used to estimate the map of adequate habitat of the species. See details in the text. [file peerj-09-11925-s002.docx]

**Supplemental Table S2:**

**Land use categories selected to elaborate the habitat map of Dupont’s Lark in Spain.**

CORINE land use categories that accounted for 95% of Dupont’s Lark post-2000 observations, and were used to estimate the map of adequate habitat of the species. See details in the text.

| CORINE ID | Description |
| --- | --- |
| 323 | Sclerophyllous vegetation |
| 321 | Natural grasslands |
| 333 | Sparsely vegetated areas |
| 243 | Land principally occupied by agriculture, with significant areas of natural vegetation |
| 211 | Non-irrigated arable land |
