# Supplementary material for: Connectivity in Spanish metapopulation of Dupont’s lark may be maintained by dispersal over medium-distance range and stepping stones
Source: PeerJ. 2021 Aug 19;9:e11925. doi: 10.7717/peerj.11925 (PMC8380426; doi:10.7717/peerj.11925)
Supplement: Supplemental Information 3 — List of populations and subpopulations of the Iberian metapopulation of the Dupont’s Lark. The metapopulation is formed by 24 populations and 100 subpopulations. For each of them, it is indicated the province; date of the last available census information; number of territorial males; habitat area (HS, in ha); mean habitat quality (HQ, providing the value of probability of presence in the Maxent model, see García-Antón et al., 2019); number of habitat patches within the subpopulation; and AHS (Available Habitat Surface) value (see main text for more information). Complete cartography is provided in Supplemental Data S1. [file peerj-09-11925-s003.docx]

| Population | ID | Subpopulation | Prov. | Last info. | Nº males | HS (ha) | HQ (prob) | Nº patches | AHS |
| --- | --- | --- | --- | --- | --- | --- | --- | --- | --- |
| 1. Arribes del Duero Oeste | 1 | Fariza | ZAMORA | 2008 | 10 | 2010.12 | 0.07 | 1 | 140.71 |
| 2. Sierra de la Culebra | 2 | Donadillo | ZAMORA | 2005 | 1 | 1256.3 | 0.09 | 2 | 56.53 |
|  | 3 | Villanueva de Valrojo | ZAMORA | 2008 | 1 | 520.01 | 0.12 | 4 | 15.6 |
|  | 4 | Tábara | ZAMORA | 2008 | 13 | 2058.35 | 0.13 | 2 | 133.79 |
|  | 5 | Vegalatrave | ZAMORA | 2008 | 1 | 1472.34 | 0.07 | 2 | 51.53 |
| 3. Arribes del Duero Este | 6 | Villaseco del Pan | ZAMORA | 2008 | 20 | 2146.97 | 0.15 | 5 | 64.41 |
|  | 7 | Almaraz de Duero | ZAMORA | 2008 | 21 | 3492.2 | 0.16 | 9 | 62.08 |
| 4. Moncayo-Gómara | 8 | Velilla de la Sierra | SORIA | 2005 | 1 | 1698.25 | 0.17 | 2 | 144.35 |
|  | 9 | Arancón | SORIA | 2005 | 3 | 2032.45 | 0.29 | 1 | 589.41 |
|  | 10 | Aldealpozo | SORIA | 2006 | 6 | 1478.56 | 0.31 | 1 | 458.35 |
|  | 11 | Pozalmuro | SORIA | 2006 | 1 | 1774.53 | 0.18 | 1 | 319.42 |
|  | 12 | Pinilla del Campo | SORIA | 2011 | 3 | 4742.02 | 0.23 | 1 | 1090.66 |
|  | 13 | Dévanos | SORIA | 2009 | 4 | 888.9 | 0.25 | 6 | 37.04 |
|  | 14 | Aranda de Moncayo | ZARAGOZA | 2011 | 34 | 4421.48 | 0.46 | 10 | 203.39 |
|  | 15 | Deza | SORIA | 2003 | 1 | 1900.49 | 0.14 | 1 | 266.07 |
| 5. Ablitas | 16 | Ablitas | NAVARRA | 2015 | 21 | 2660.03 | 0.36 | 7 | 136.8 |
| 6. Bardenas | 17 | Bardenas | NAVARRA | 2018 | 70 | 5283.06 | 0.55 | 2 | 1452.84 |
| 7. Campo de borja | 18 | Talamantes | ZARAGOZA | 2017 | 1 | 423.13 | 0.33 | 5 | 27.93 |
| 8. Tardienta | 19 | Tardienta | HUESCA | 2007 | 10 | 2824.62 | 0.32 | 3 | 301.29 |
| 9. Bajo Cinca | 20 | Ballobar | HUESCA | 2017 | 12 | 7688.6 | 0.16 | 2 | 615.09 |
| 10. Alfés | 21 | Alfés | LÉRIDA | 2018 | 7 | 2020.88 | 0.02 | 1 | 40.42 |
| 11. S. Ibérico - V. del Ebro | 22 | Páramo de Corcos | BURGOS | 2017 | 10 | 3533.02 | 0.26 | 3 | 306.2 |
|  | 23 | Hoces del Riaza | SEGOVIA | 2018 | 56 | 2194.35 | 0.38 | 16 | 52.12 |
|  | 24 | Los Castros | SEGOVIA | 2017 | 4 | 1833.74 | 0.43 | 2 | 394.25 |
|  | 25 | Hoces del Duratón | SEGOVIA | 2018 | 62 | 5411.84 | 0.51 | 10 | 276 |
|  | 26 | Cenegro | SORIA | 2006 | 4 | 1894.76 | 0.42 | 1 | 795.8 |
|  | 27 | Atauta | SORIA | 2006 | 7 | 1962.57 | 0.16 | 4 | 78.5 |
|  | 28 | Las Fraguas | SORIA | 2011 | 8 | 2207.95 | 0.41 | 3 | 301.75 |
|  | 29 | Nafría la Llana | SORIA | 2014 | 6 | 2198.73 | 0.37 | 1 | 813.53 |
|  | 30 | Fuentelárbol | SORIA | 2006 | 1 | 1690.12 | 0.33 | 1 | 557.74 |
|  | 31 | Brías | SORIA | 2006 | 11 | 3075.33 | 0.38 | 6 | 194.77 |
|  | 32 | Altos de Barahona | SORIA | 2017 | 777 | 49150.55 | 0.38 | 67 | 278.76 |
|  | 33 | Hiendelaencina | GUADALAJARA | 2017 | 8 | 1367.66 | 0.26 | 6 | 59.27 |
|  | 34 | Conquezuela | SORIA | 2017 | 3 | 1660.25 | 0.26 | 4 | 107.92 |
|  | 35 | Sigüenza | GUADALAJARA | 2017 | 61 | 4537.57 | 0.23 | 15 | 69.58 |
|  | 36 | Layna | SORIA | 2017 | 526 | 40048.52 | 0.33 | 55 | 240.29 |
|  | 37 | La Torresaviñán | GUADALAJARA | 2017 | 16 | 5358.77 | 0.35 | 2 | 937.78 |
|  | 38 | Hortezuela de Océn | GUADALAJARA | 2017 | 3 | 1171.81 | 0.24 | 3 | 93.74 |
|  | 39 | Riba de Saelices | GUADALAJARA | 2017 | 13 | 4763.63 | 0.26 | 6 | 206.42 |
|  | 40 | Cueva de la Hoz | GUADALAJARA | 2017 | 1 | 293.49 | 0.16 | 2 | 23.48 |
|  | 41 | Maranchón | GUADALAJARA | 2017 | 1 | 2652.74 | 0.46 | 3 | 406.75 |
|  | 42 | Alhama de Aragón | ZARAGOZA | 2009 | 2 | 116.79 | 0.36 | 2 | 21.02 |
|  | 43 | Milmarcos-Llumes | GUADALAJARA | 2016 | 36 | 5751.62 | 0.46 | 18 | 146.99 |
|  | 44 | Paramera de Molina | GUADALAJARA | 2017 | 161 | 17905.63 | 0.47 | 8 | 1051.96 |
|  | 45 | Torralba de los Frailes | TERUEL | 2007 | 20 | 4671.77 | 0.39 | 1 | 1821.99 |
|  | 46 | Calatayud | ZARAGOZA | 2017 | 87 | 5206.42 | 0.55 | 10 | 286.35 |
|  | 47 | Campo Romanos | ZARAGOZA | 2016 | 15 | 2462.57 | 0.44 | 1 | 1083.53 |
|  | 48 | Segura de los Baños | TERUEL | 2017 | 156 | 17293.59 | 0.58 | 31 | 323.56 |
|  | 49 | Lechago | TERUEL | 2007 | 3 | 794.46 | 0.57 | 6 | 75.47 |
|  | 50 | Cutanda | TERUEL | 2007 | 16 | 1474.49 | 0.46 | 11 | 61.66 |
|  | 51 | Castilnovo | GUADALAJARA | 2017 | 5 | 1772.6 | 0.3 | 2 | 265.89 |
|  | 52 | Río Gallo | GUADALAJARA | 2017 | 92 | 9514.57 | 0.51 | 12 | 404.37 |
|  | 53 | Blancas | TERUEL | 2017 | 175 | 13451.22 | 0.56 | 2 | 3766.34 |
|  | 54 | Puerto Bañón | TERUEL | 2007 | 4 | 1027.7 | 0.51 | 3 | 174.71 |
|  | 55 | Altiplano de Teruel | TERUEL | 2018 | 346 | 37798.31 | 0.54 | 55 | 371.11 |
|  | 56 | Villar del Salz | TERUEL | 2007 | 13 | 2738.05 | 0.55 | 8 | 188.24 |
|  | 57 | Aguatón | TERUEL | 2007 | 19 | 1865 | 0.51 | 3 | 317.05 |
|  | 58 | Orihuela del Tremedal | TERUEL | 2009 | 1 | 1786.63 | 0.56 | 1 | 1000.51 |
|  | 59 | Pozondón | TERUEL | 2011 | 38 | 4494.91 | 0.56 | 4 | 629.29 |
|  | 60 | Gea de Albarracín | TERUEL | 2013 | 3 | 783.06 | 0.65 | 7 | 72.71 |
|  | 61 | Celadas Oeste | TERUEL | 2007 | 2 | 1946.91 | 0.37 | 2 | 360.18 |
|  | 62 | Celadas Este | TERUEL | 2007 | 11 | 3392.83 | 0.43 | 3 | 486.31 |
|  | 63 | Valdecebro | TERUEL | 2017 | 1 | 622.18 | 0.48 | 7 | 42.66 |
|  | 64 | Allepuz | TERUEL | 2007 | 7 | 1131.4 | 0.58 | 7 | 93.74 |
|  | 65 | Alagón | ZARAGOZA | 2005 | 1 | 287.59 | 0.42 | 4 | 30.2 |
|  | 66 | Utebo | ZARAGOZA | 2014 | 1 | 193.28 | 0.36 | 5 | 13.92 |
|  | 67 | Juslibol | ZARAGOZA | 2014 | 2 | 893.03 | 0.55 | 2 | 245.58 |
|  | 68 | Lumpiaque | ZARAGOZA | 2017 | 2 | 1851.92 | 0.39 | 3 | 240.75 |
|  | 69 | Val de Urrea | ZARAGOZA | 2016 | 75 | 3674.88 | 0.54 | 14 | 141.75 |
|  | 70 | Longares-Mezalocha | ZARAGOZA | 2011 | 12 | 1830.42 | 0.44 | 8 | 100.67 |
|  | 71 | Cuarte de Huerva | ZARAGOZA | 2007 | 5 | 924.08 | 0.56 | 5 | 103.5 |
|  | 72 | Belchite | ZARAGOZA | 2007 | 234 | 24271.96 | 0.51 | 13 | 952.21 |
|  | 73 | Monegros | ZARAGOZA | 2017 | 97 | 19845.62 | 0.52 | 2 | 5159.86 |
|  | 74 | Gelsa | ZARAGOZA | 2013 | 12 | 3699.92 | 0.52 | 2 | 961.98 |
|  | 75 | Laguna de Pito | ZARAGOZA | 2005 | 1 | 1845.42 | 0.17 | 1 | 313.72 |
|  | 76 | Bujaraloz | ZARAGOZA | 2008 | 1 | 1951.57 | 0.09 | 1 | 175.64 |
|  | 77 | Alforque | ZARAGOZA | 2008 | 2 | 812.41 | 0.44 | 1 | 357.46 |
|  | 78 | Azaila | TERUEL | 2008 | 3 | 2564.05 | 0.35 | 4 | 224.35 |
|  | 79 | Vinaceite | TERUEL | 2008 | 1 | 1638.54 | 0.16 | 2 | 131.08 |
|  | 80 | Lagata | ZARAGOZA | 2006 | 1 | 1461.34 | 0.47 | 2 | 343.41 |
|  | 81 | Lécera | ZARAGOZA | 2017 | 28 | 2399.91 | 0.54 | 1 | 1295.95 |
|  | 82 | Albalate del Arzobispo | TERUEL | 2007 | 36 | 3164.97 | 0.44 | 2 | 696.29 |
| 12. Ejulve | 83 | Molinos | TERUEL | 2017 | 27 | 2223.84 | 0.55 | 14 | 87.37 |
| 13. Uclés-Saelices | 84 | Saelices | CUENCA | 2018 | 2 | 5891.05 | 0.09 | 3 | 176.73 |
| 14. Zafra de Záncara | 85 | Zafra de Záncara | CUENCA | 2018 | 3 | 1740.45 | 0.08 | 3 | 46.41 |
| 15. Valeria | 86 | Valeria | CUENCA | 2018 | 16 | 2964.45 | 0.11 | 5 | 65.22 |
| 16. Carboneras de Guadazaón | 87 | Carboneras de Guadazaón | CUENCA | 2018 | 6 | 2738.44 | 0.23 | 1 | 629.84 |
|  | 88 | Cardenete | CUENCA | 2018 | 6 | 452.6 | 0.12 | 5 | 10.86 |
| 17. Ademuz | 89 | Moya | CUENCA | 2018 | 55 | 4805.49 | 0.28 | 10 | 134.55 |
|  | 90 | Ademuz | VALENCIA | 2018 | 56 | 4278.63 | 0.44 | 22 | 85.57 |
|  | 91 | Sierra de Javalambre | TERUEL | 2007 | 75 | 1170.04 | 0.55 | 6 | 107.25 |
| 18. Hoya Gonzalo | 92 | Hoya Gonzalo | ALBACETE | 2018 | 3 | 3215.46 | 0.22 | 3 | 235.8 |
| 19. Yecla | 93 | Herrada del Manco | MURCIA | 2017 | 1 | 732.84 | 0.11 | 7 | 40.31 |
|  | 94 | Moratillas | MURCIA | 2016 | 0 | 635.48 | 0.07 | 2 | 6.35 |
| 20. Cieza | 95 | Sierra del Picarcho | MURCIA | 2017 | 10 | 2062.32 | 0.22 | 6 | 75.62 |
| 21. Padul | 96 | Padul | GRANADA | 2015 | 2 | 1711.96 | 0.13 | 8 | 27.82 |
| 22. Sierra de Gádor | 97 | LLano de Los Brincos-La Mota | ALMERÍA | 2016 | 1 | 555.18 | 0.21 | 10 | 11.66 |
| 23. Tabernas-Sorbas | 98 | Los Sebastianes | ALMERÍA | 2017 | 3 | 2068.66 | 0.44 | 4 | 227.55 |
|  | 99 | Karst de Sorbas | ALMERÍA | 2016 | 4 | 946.1 | 0.29 | 3 | 91.46 |
| 24. Cabo de Gata | 100 | Las Amoladeras | ALMERÍA | 2017 | 12 | 2155.72 | 0.21 | 1 | 452.7 |
