# Supplementary material for: Connectivity in Spanish metapopulation of Dupont’s lark may be maintained by dispersal over medium-distance range and stepping stones
Source: PeerJ. 2021 Aug 19;9:e11925. doi: 10.7717/peerj.11925 (PMC8380426; doi:10.7717/peerj.11925)
Supplement: Supplemental Information 4 — The Equivalent Connectivity Index represents the global connectivity of the metapopulation. Both the movement threshold and the presence of stepping stones generate increments in connectivity, with a stronger effect of the latter. [file peerj-09-11925-s004.docx]

| Movements | EC without s. stones | EC with s. stones |
| --- | --- | --- |
| Short distance movements (5 km) | 8935.65 (scenario 1) | 14560.55 (scenario 4) |
| Medium distance movements (20 km) | 11529.18 (scenario 2) | 24340.81 (scenario 5) |
| Long distance movements (100 km) | 21956.86 (scenario 3) | 46319.15 (scenario 6) |
