# Supplementary material for: Connectivity in Spanish metapopulation of Dupont’s lark may be maintained by dispersal over medium-distance range and stepping stones
Source: PeerJ. 2021 Aug 19;9:e11925. doi: 10.7717/peerj.11925 (PMC8380426; doi:10.7717/peerj.11925)

Population nº 1: Arribes del Duero Oeste. 1 subpopulation: Fariza.

Population nº 2: Sierra de la Culebra. 4 subpopulations: Donadillo, Villanueva de Valrojo, Tábara, Vegalatrave.

Population nº 3: Arribes del Duero Este. 2 subpopulations: Villaseco del Pan, Almaraz de Duero.

Subpopulations of recent extinction (post-2000): Sierra de Cantadores, Páramo de Castañeda.

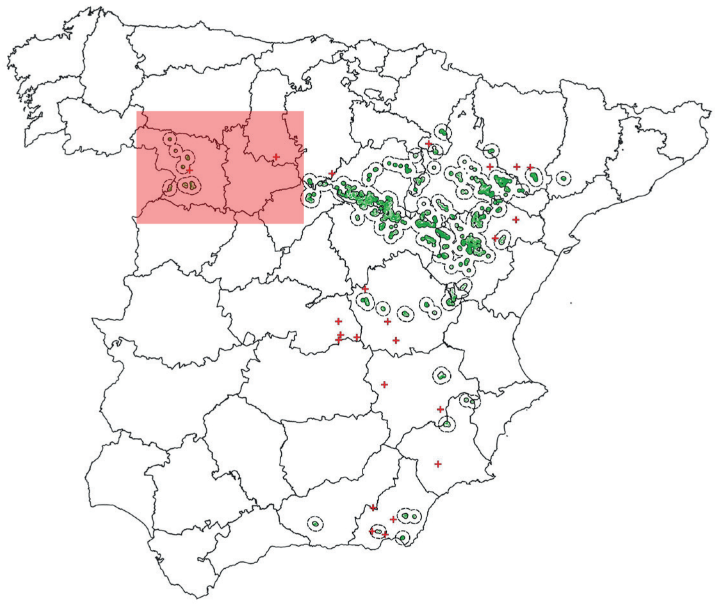

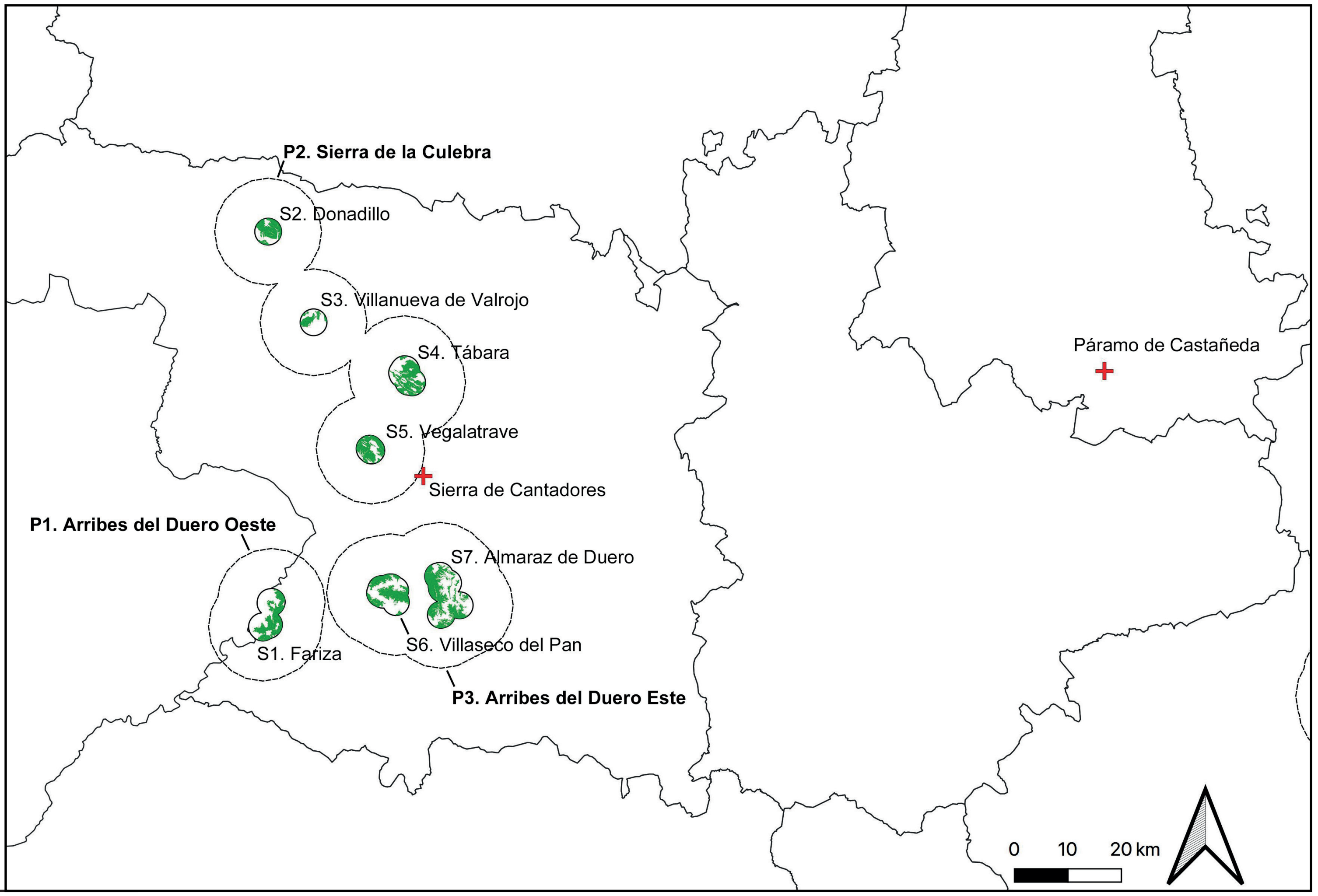

Population nº 4: Moncayo-Gómara. 8 subpopulations: Velilla de la Sierra, Arancón, Aldealpozo, Pozalmuro, Pinilla del Campo, Dévanos, Aranda de Moncayo, Deza.

Population nº 5: Ablitas. 1 subpopulation: Ablitas.

Population nº 6: Bardenas. 1 subpopulation: Bardenas.

Population nº 7: Campo de borja. 1 subpopulation: Talamantes.

Subpopulations of recent extinction (post-2000): Balsa del Pulguer.

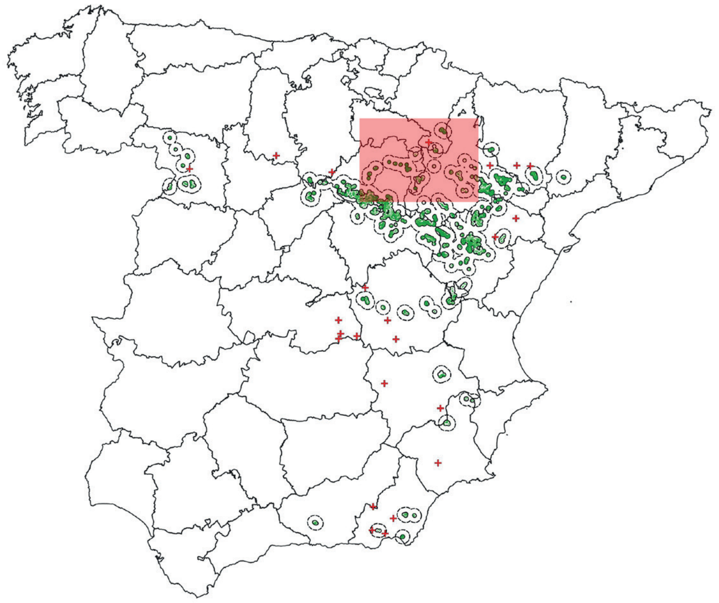

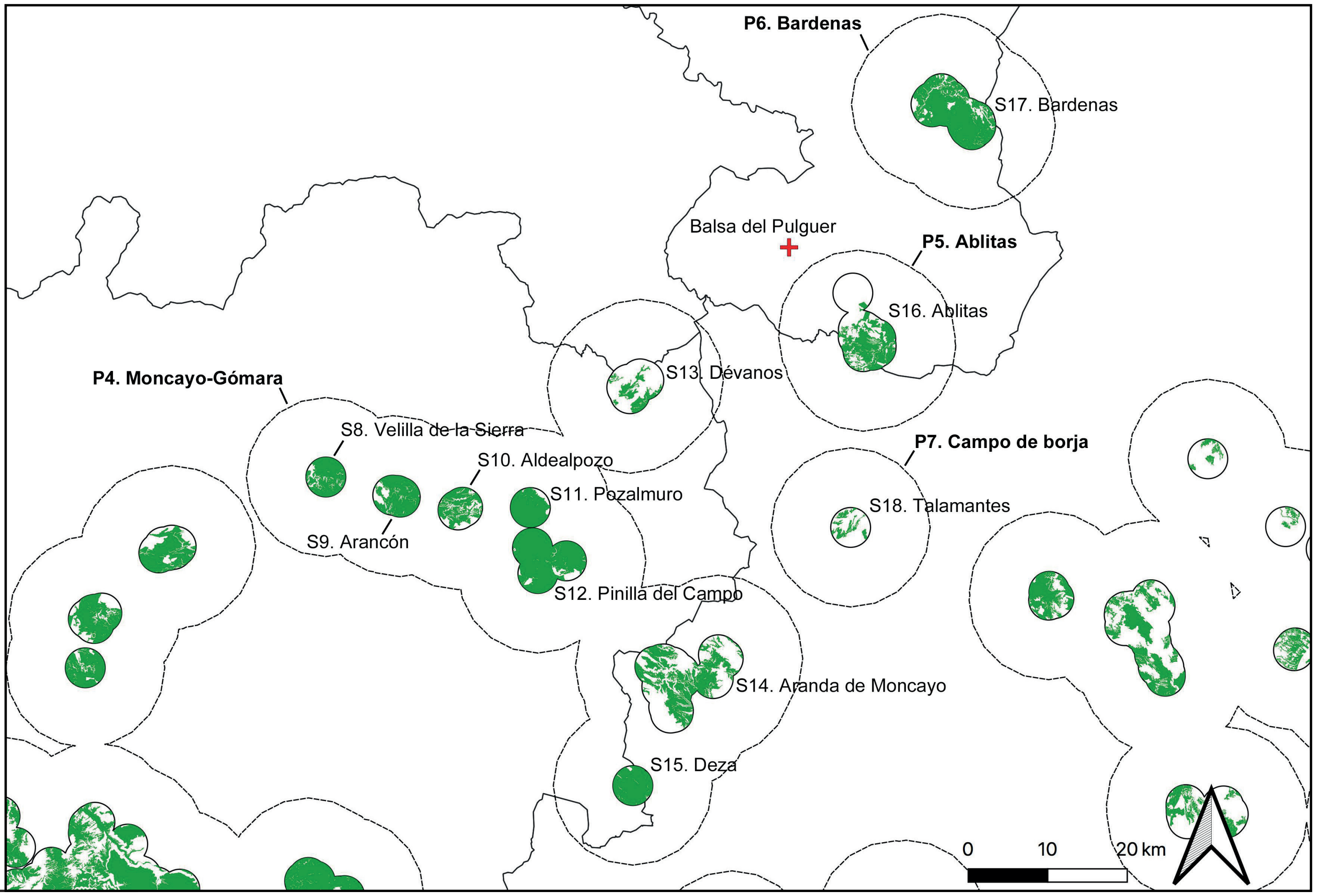

Population nº 8: Tardienta. 1 subpopulation: Tardienta.

Population nº 9: Bajo Cinca. 1 subpopulation: Ballobar.

Population nº 10: Alfés. 1 subpopulation: Alfés.

Subpopulations of recent extinction (post-2000): Perdiguera, Albalatillo, Alcolea de Cinca.

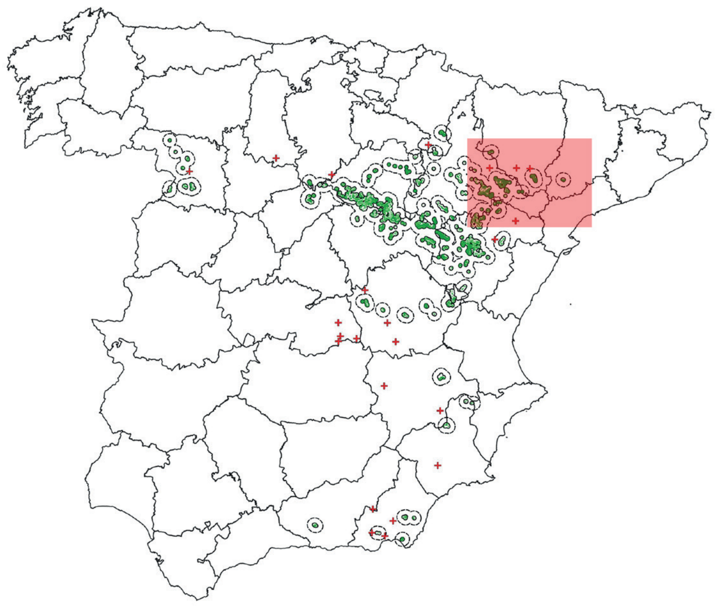

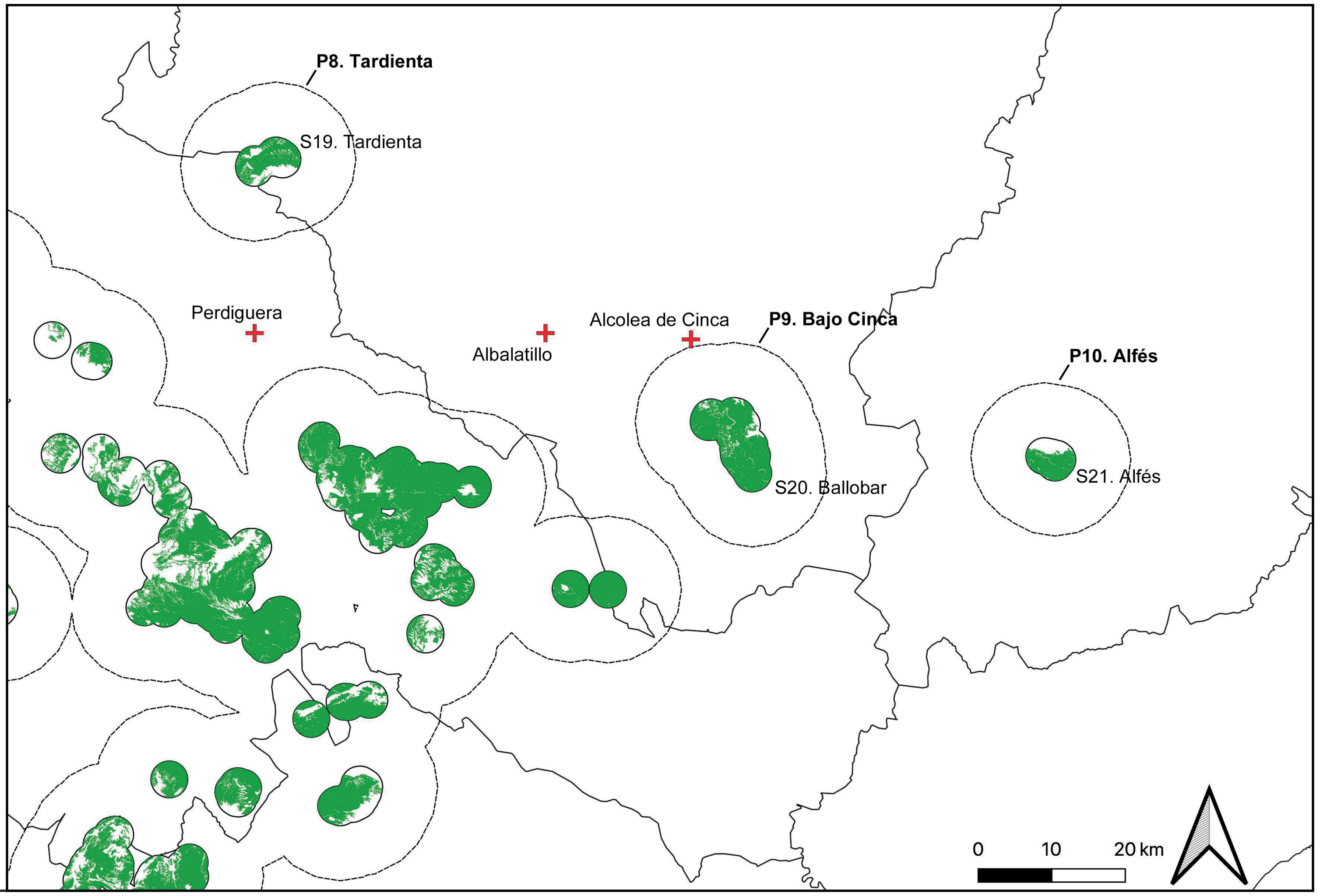

Population nº 11: S. Ibérico - V. del Ebro. 61 subpopulations: Páramo de Corcos, Hoces del Riaza, Los Castros, Hoces del Duratón, Cenegro, Atauta, Las Fraguas, Nafría la Llana, Fuentelárbol, Brías, Altos de Barahona, Hiendelaencina, Conquezuela, Sigüenza, Layna, La Torresaviñán, Hortezuela de Océn, Riba de Saelices, Cueva de la Hoz, Maranchón, [continues in the next map].

Subpopulations of recent extinction (post-2000): Casanova.

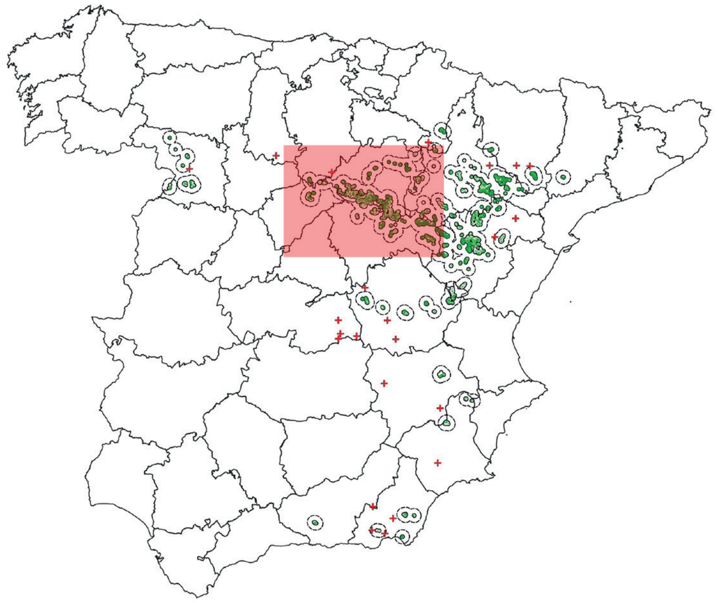

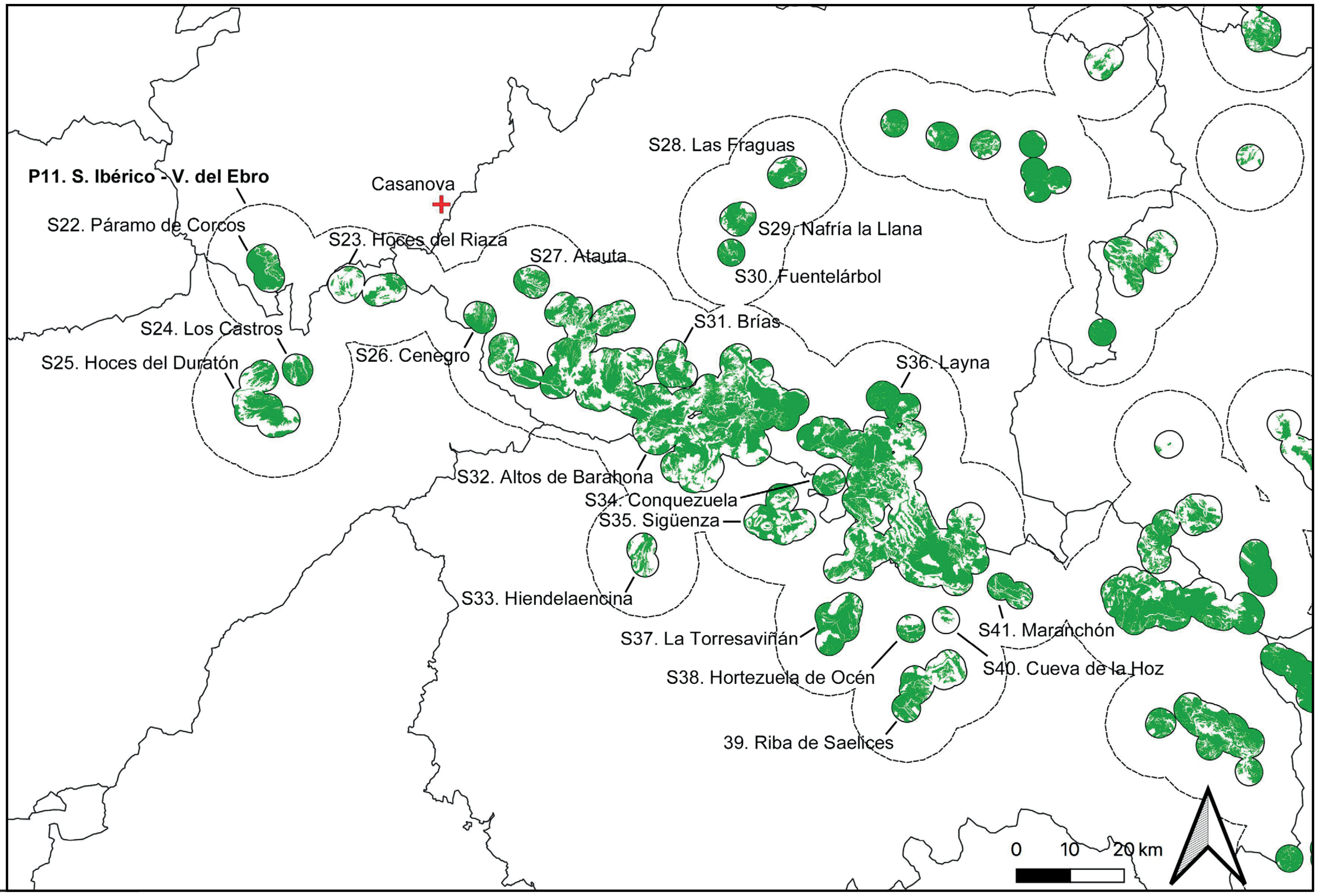

Population nº 11: S. Ibérico - V. del Ebro. 61 subpopulations: Alhama de Aragón, Milmarcos-Llumes, Paramera de Molina, Torralba de los Frailes, Calatayud, Campo Romanos, Segura de los Baños, Lechago, Cutanda, Castilnovo, Río Gallo, Blancas, Puerto Bañón, Altiplano de Teruel, Villar del Salz, Aguatón, Orihuela del Tremedal, Pozondón, Gea de Albarracín, Celadas Oeste, Celadas Este Valdecebro, Allepuz, [continues in the next map].

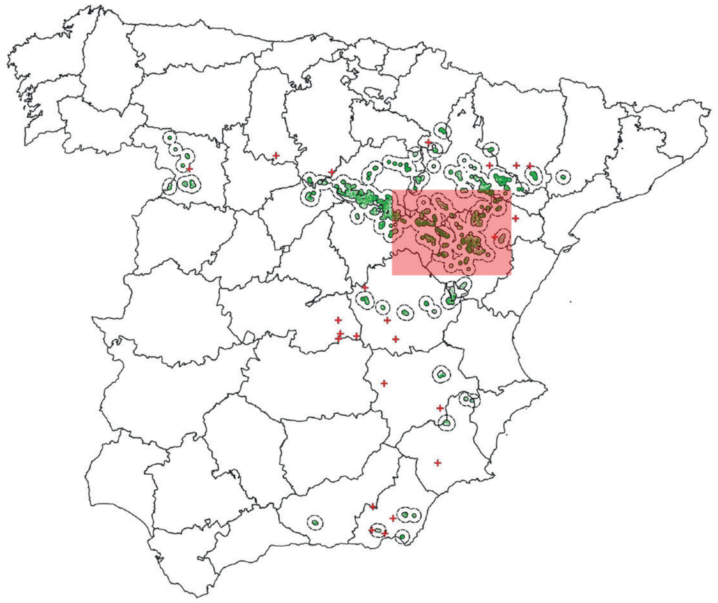

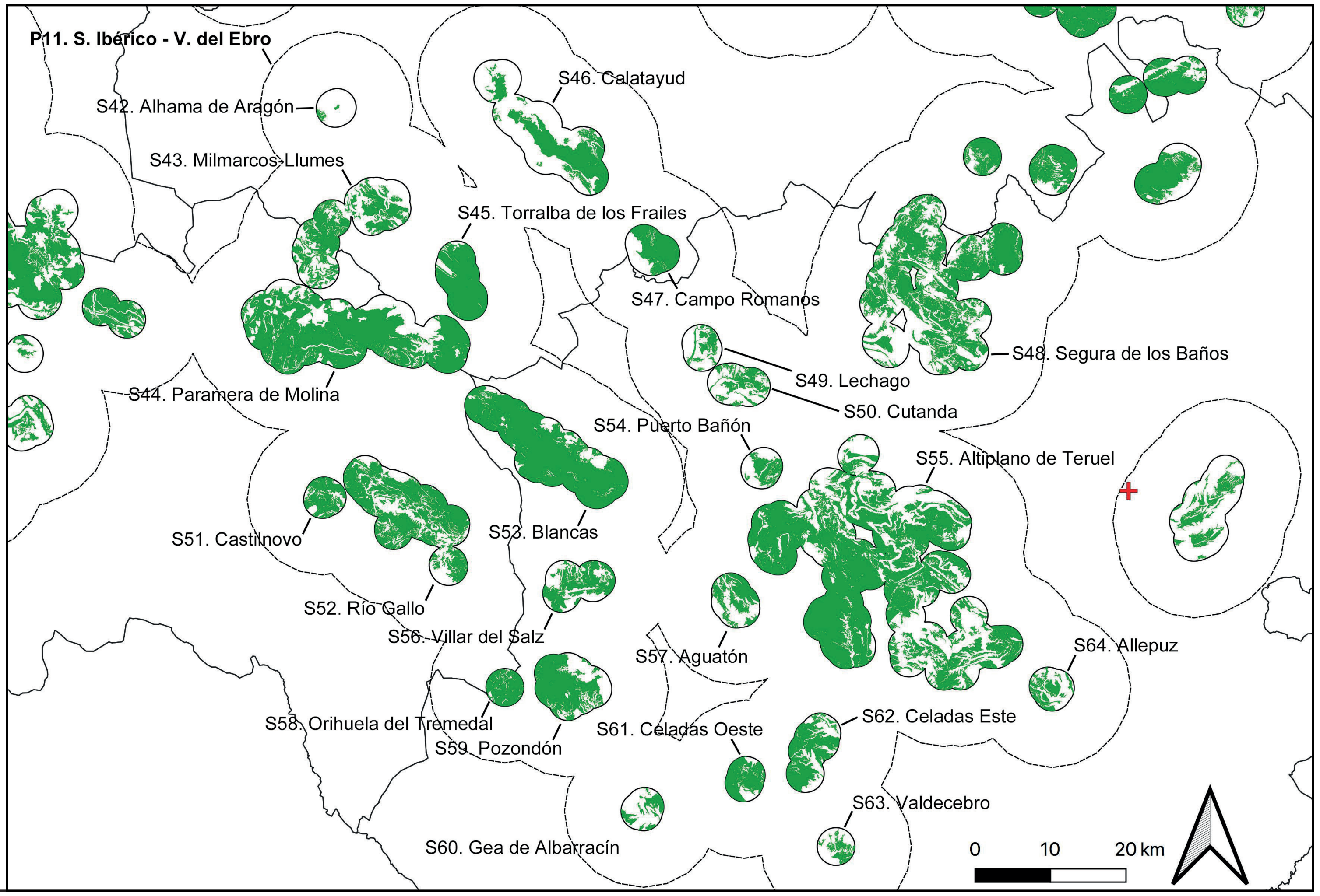

Population nº 11: S. Ibérico - V. del Ebro. 61 subpopulations: Alagón, Utebo, Juslibol, Lumpiaque, Val de Urrea, Longares-Mezalocha, Cuarte de Huerva, Belchite, Monegros, Gelsa, Laguna de Pito, Bujaraloz, Alforque, Azaila, Vinaceite, Lagata, Lécera, Albalate del Arzobispo.

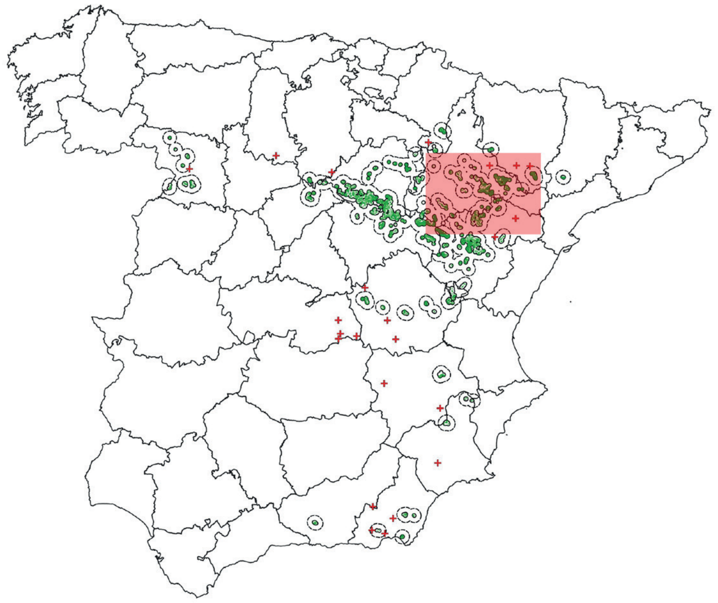

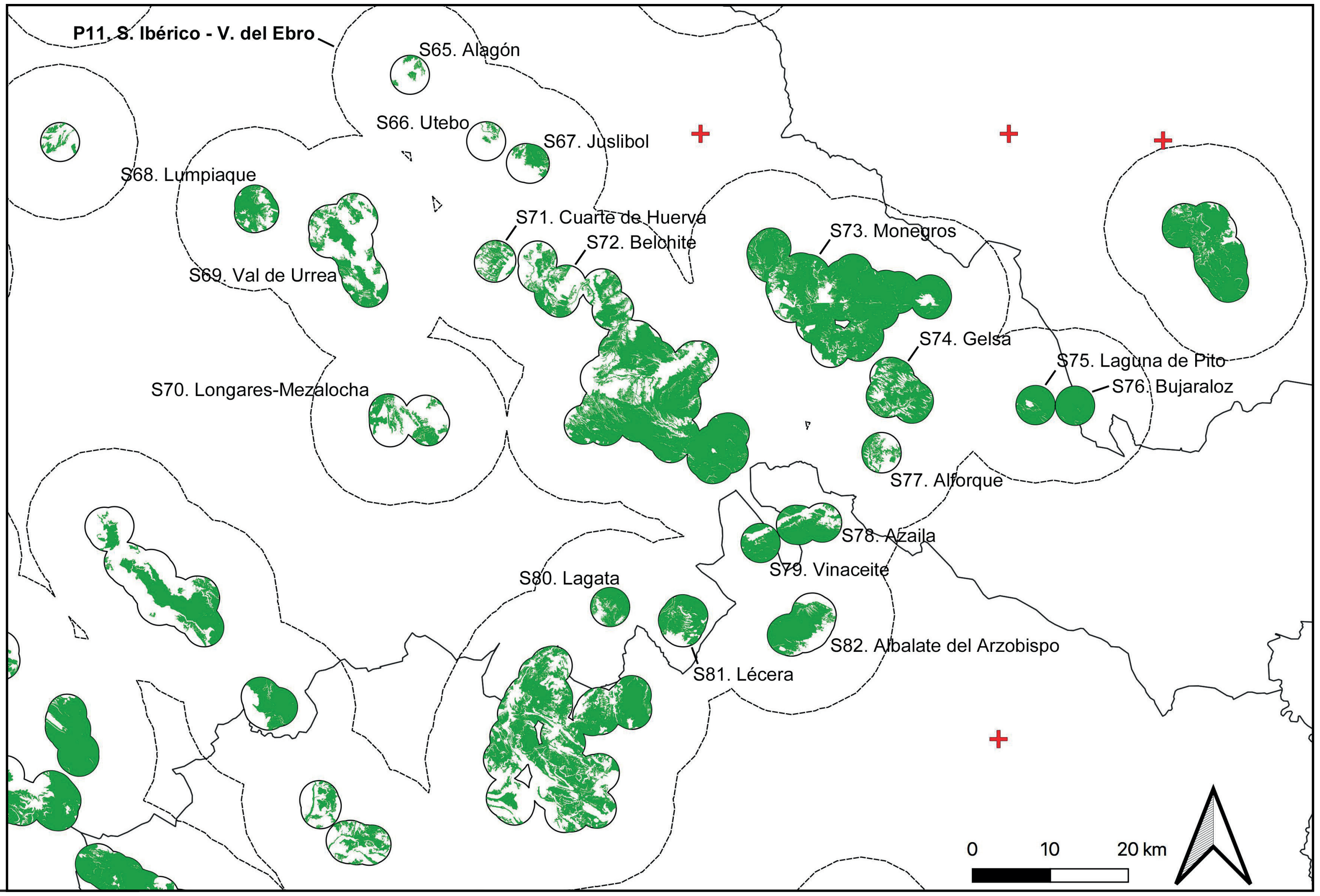

Population nº 12: Ejulve. 1 subpopulation: Molinos.

Subpopulations of recent extinction (post-2000): Gargallo-Ejulve, Alcañiz.

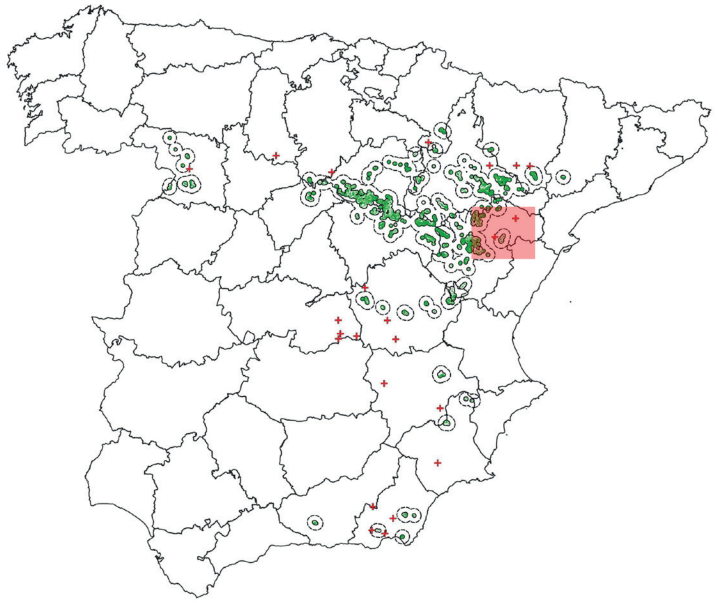

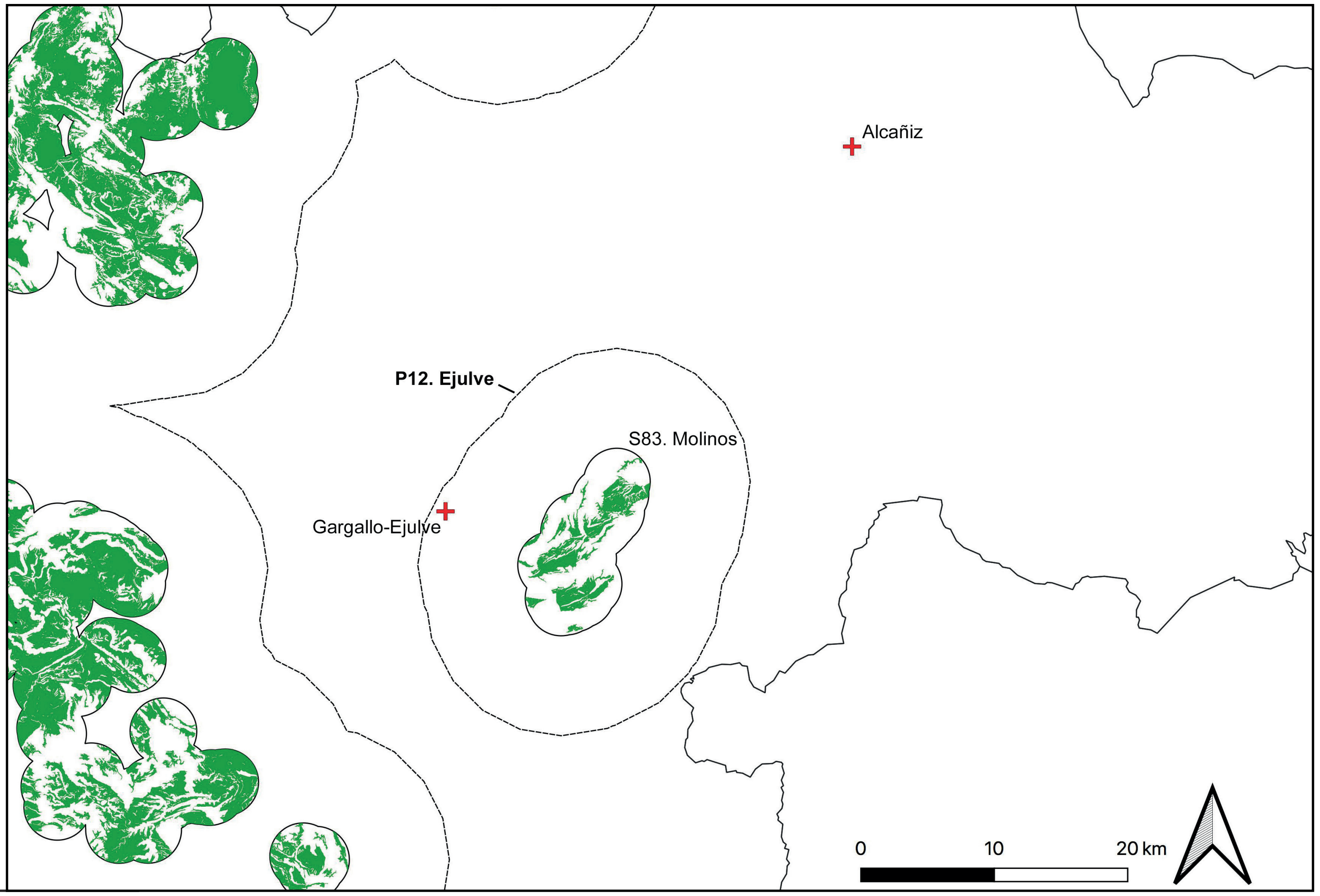

Population nº 13: Uclés-Saelices. 1 subpopulation: Saelices.

Population nº 14: Zafra de Záncara. 1 subpopulation: Zafra de Záncara.

Population nº 15: Valeria. 1 subpopulation: Valeria.

Population nº 16: Carboneras de Guadazaón. 2 subpopulations: Carboneras de Guadazaón, Cardenete.

Population nº 17: Ademuz. 3 subpopulations: Moya, Ademuz, Sierra de Javalambre.

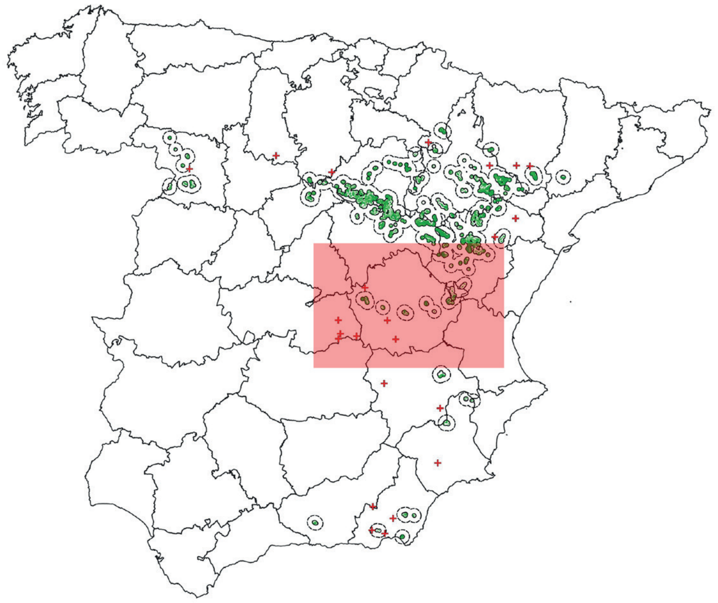

Subpopulations of recent extinction (post-2000): Lillo, Quero, Pastrana, Saceda-Trasierra, Los Tomillares, La Hinojosa, Villar de Cantos.

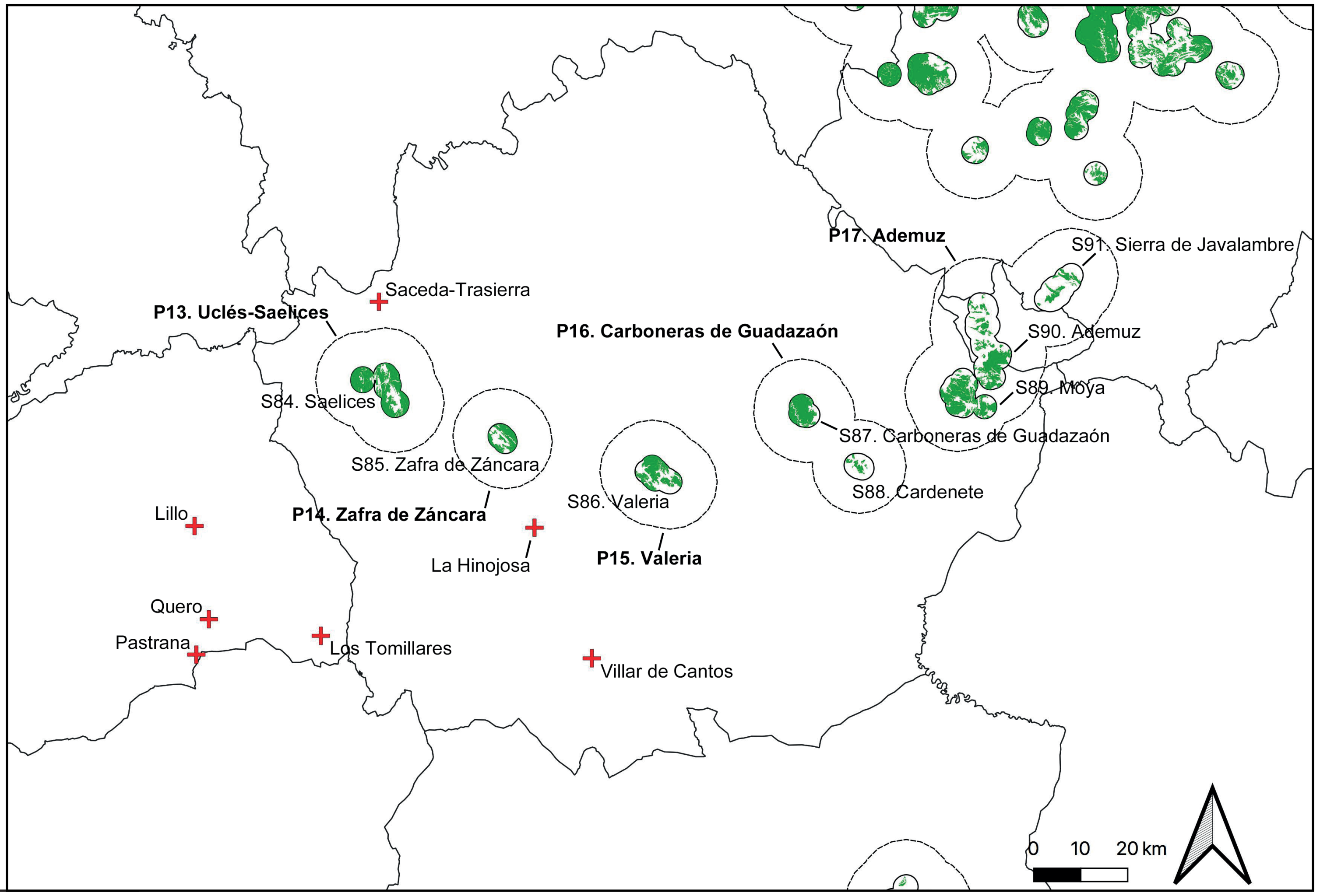

Population nº 18: Hoya Gonzalo. 1 subpopulation: Hoya Gonzalo.

Population nº 19: Yecla. 2 subpopulations: Herrada del Manco, Moratillas.

Population nº 20: Cieza. 1 subpopulation: Sierra del Picarcho.

Subpopulations of recent extinction (post-2000): El Ballestero, Las joaquinas.

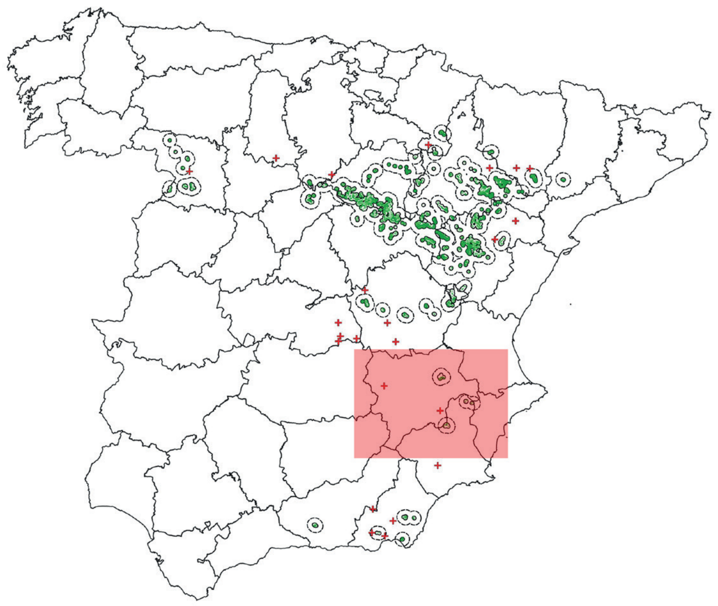

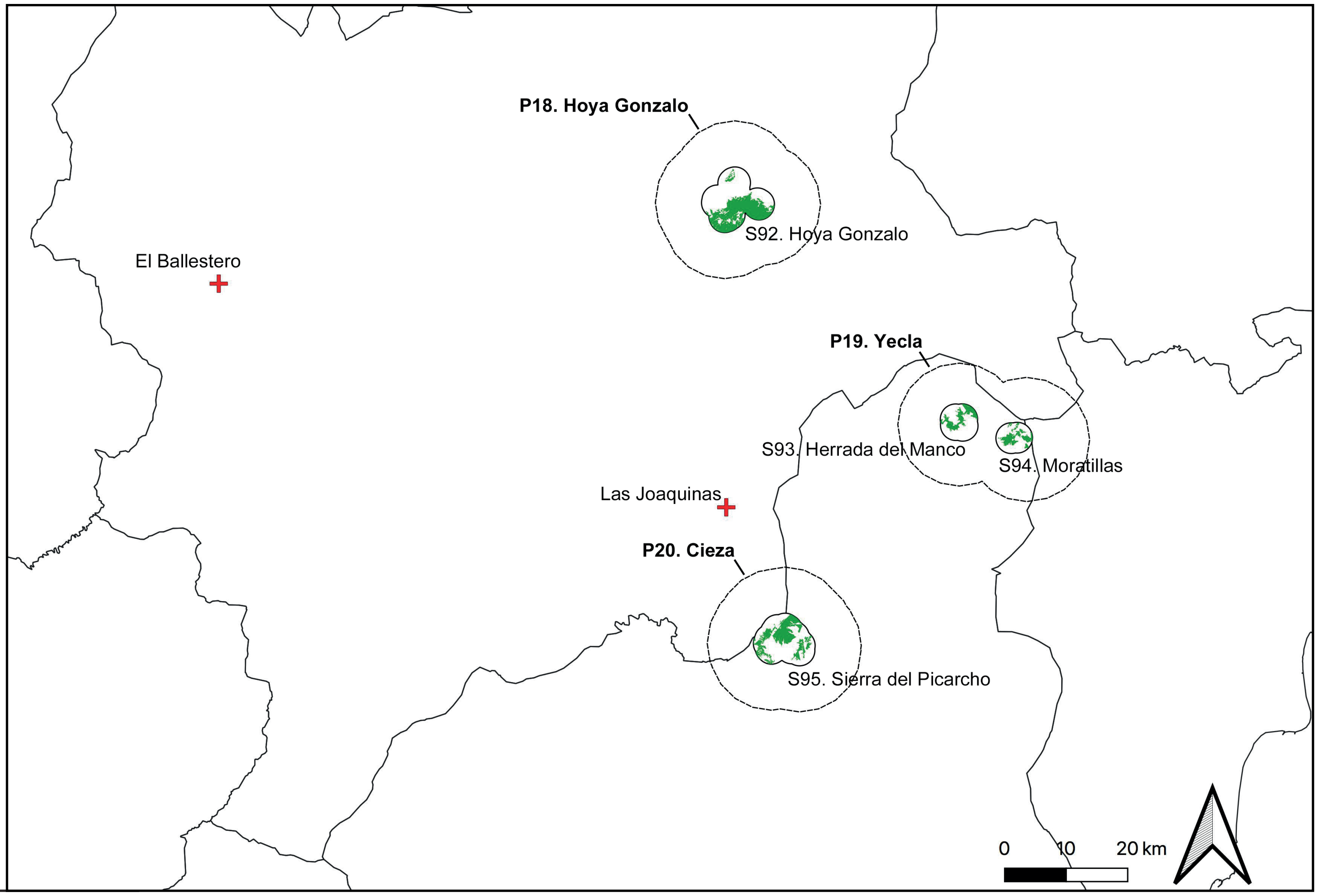

Population nº 21: Padul. 1 subpopulation: Padul.

Population nº 22: Sierra de Gádor. 1 subpopulation: LLano de Los Brincos-La Mota.

Population nº 23: Tabernas-Sorbas. 2 subpopulations: Los Sebastianes, Karst de Sorbas.

Population nº 24: Cabo de Gata. 1 subpopulation: Las Amoladeras.

Subpopulations of recent extinction (post-2000): Llano de la Cabras, Cerro Villegas Los Pilares, Llano de Canjayar, La Campita.

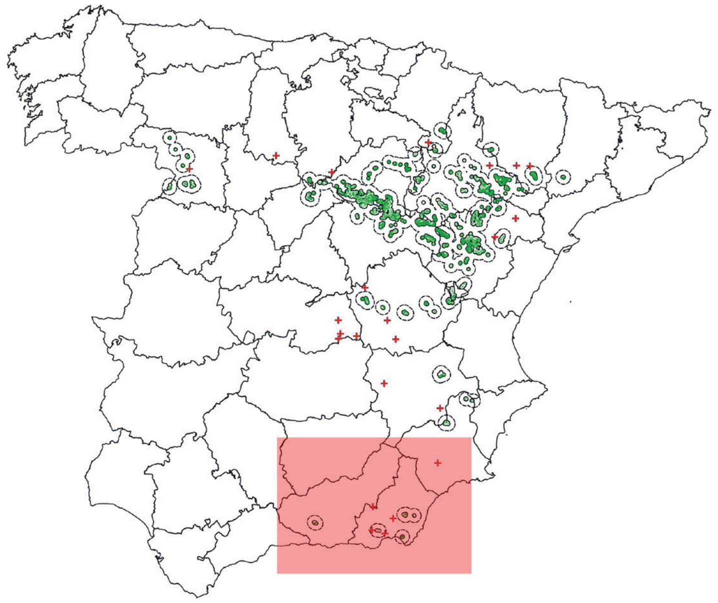

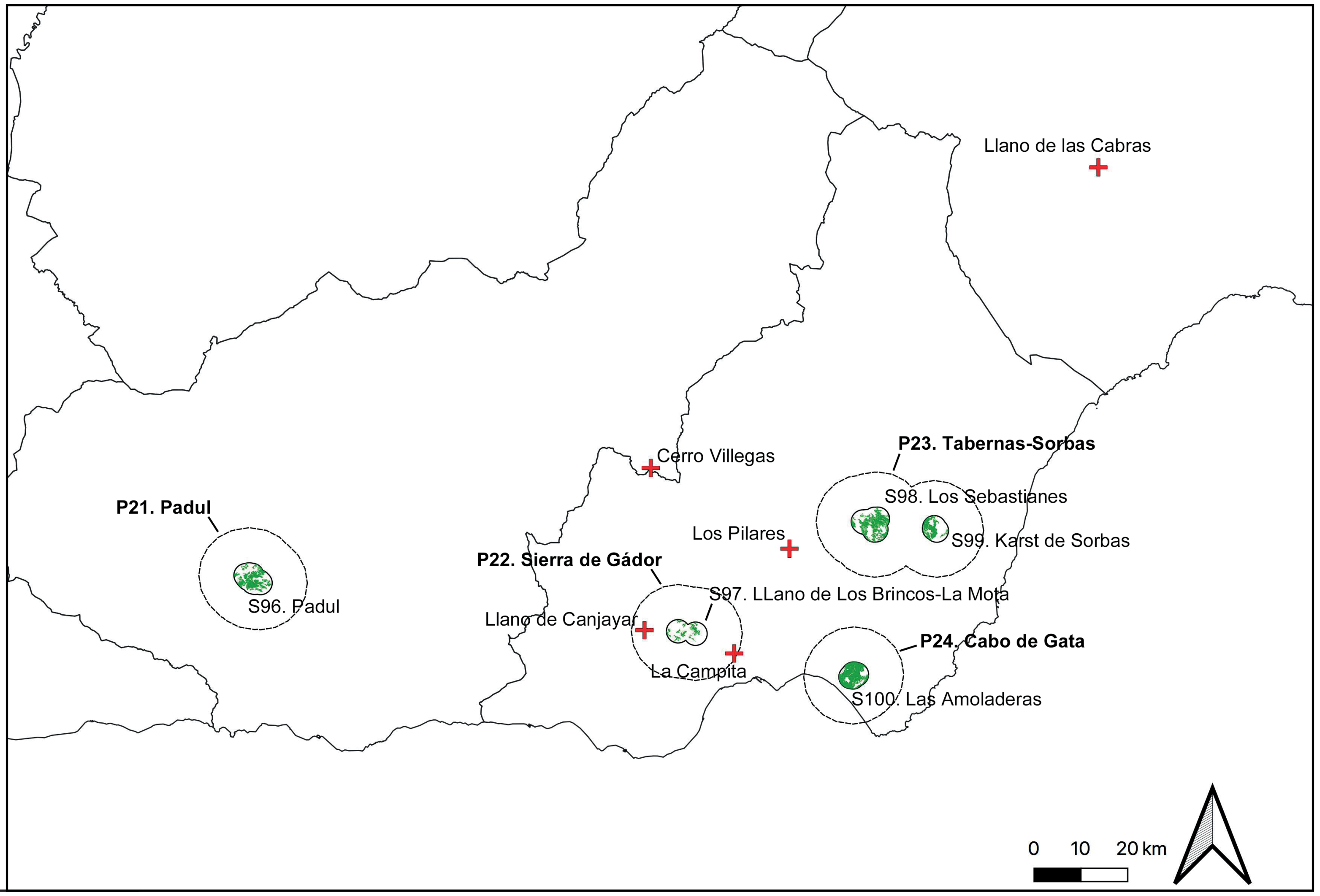

Supplement: Supplemental Information 5 — PDF file including complete cartography of the extant 24 populations (black contours) and 100 subpopulations (with habitat patches in green polygons), plus the 23 additional subpopulations of recent (post-2000) extinction (red crosses), updated to 2019. The complete list with additional information is provided in Supplemental Table S3. [file peerj-09-11925-s005.pdf]
