# Supplementary material for: Connectivity in Spanish metapopulation of Dupont’s lark may be maintained by dispersal over medium-distance range and stepping stones
Source: PeerJ. 2021 Aug 19;9:e11925. doi: 10.7717/peerj.11925 (PMC8380426; doi:10.7717/peerj.11925)

I) ABSENCE OF STEPPING STONES.

dPCintra. Internal quality of subpopulations (intra-patch connectivity), without stepping stones. The 10 most important subpopulations are indicated. Find the complete list of dPCintra values in Supplemental Data S3.

Right figure: dPCintra makes reference to the internal importance of each node and doesn't depend on spatial position. Thus, the ranking is the same for the different movement thresholds and here we only show results of scenario 1 (5 km movements, without stepping stones).

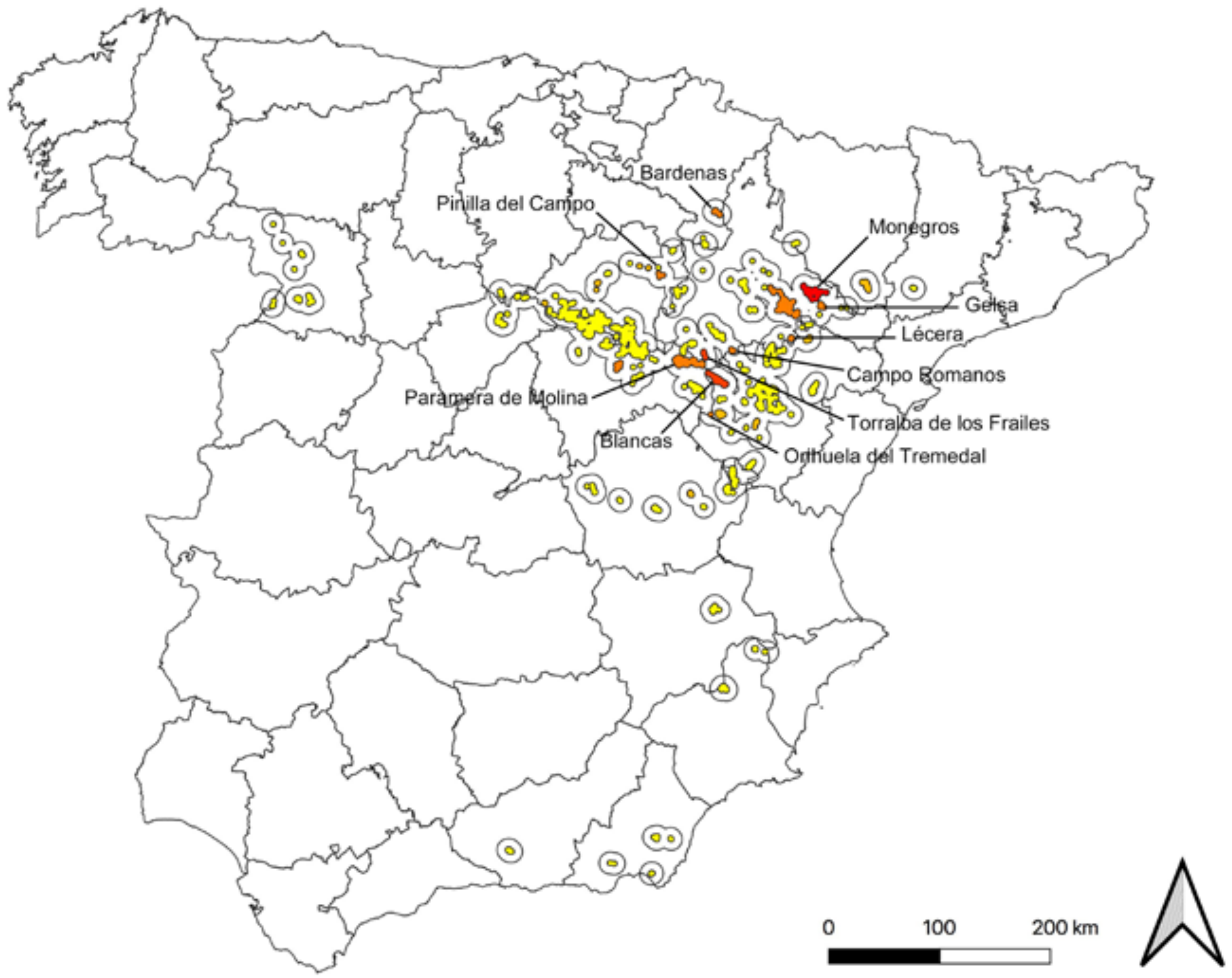

dPCflux. Degree of flow that each subpopulation generates within the network (intra-patch connectivity). The 10 most important subpopulations are indicated. Complete list of dPCflux values in Supplemental Data S3.

Down-left. 5 km movements.

Up-right. 20 km movements.

Down-right. 100 km movements.

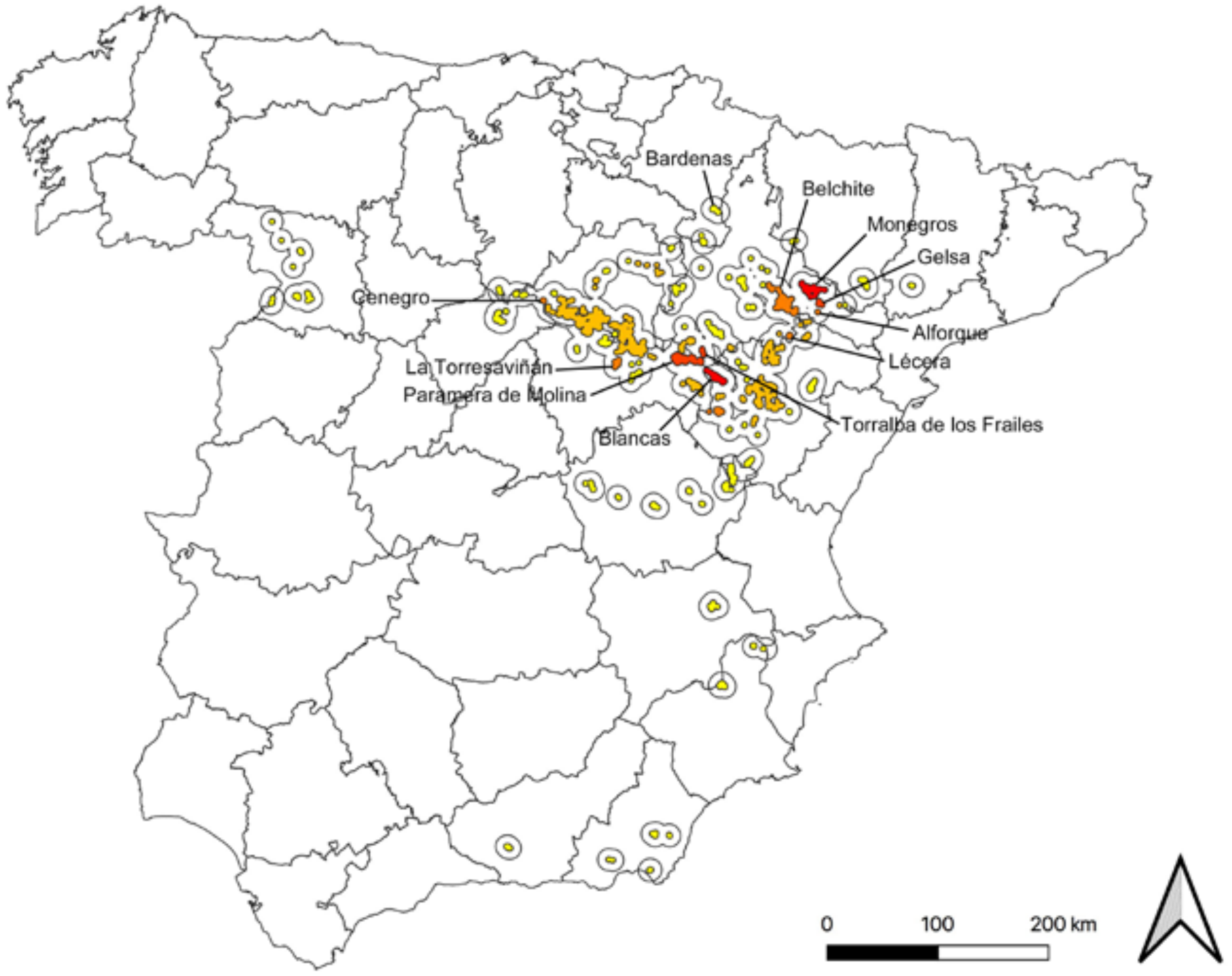

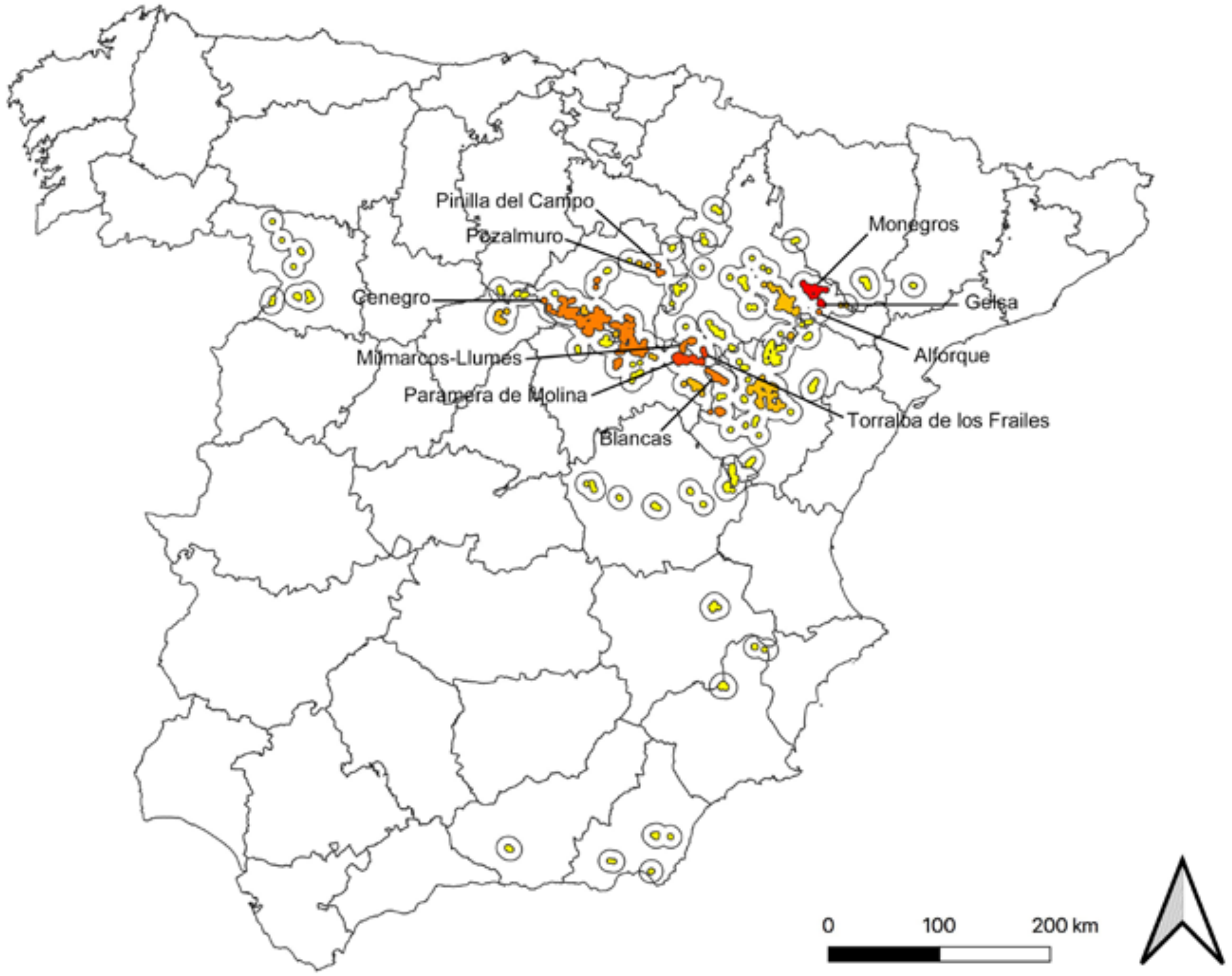

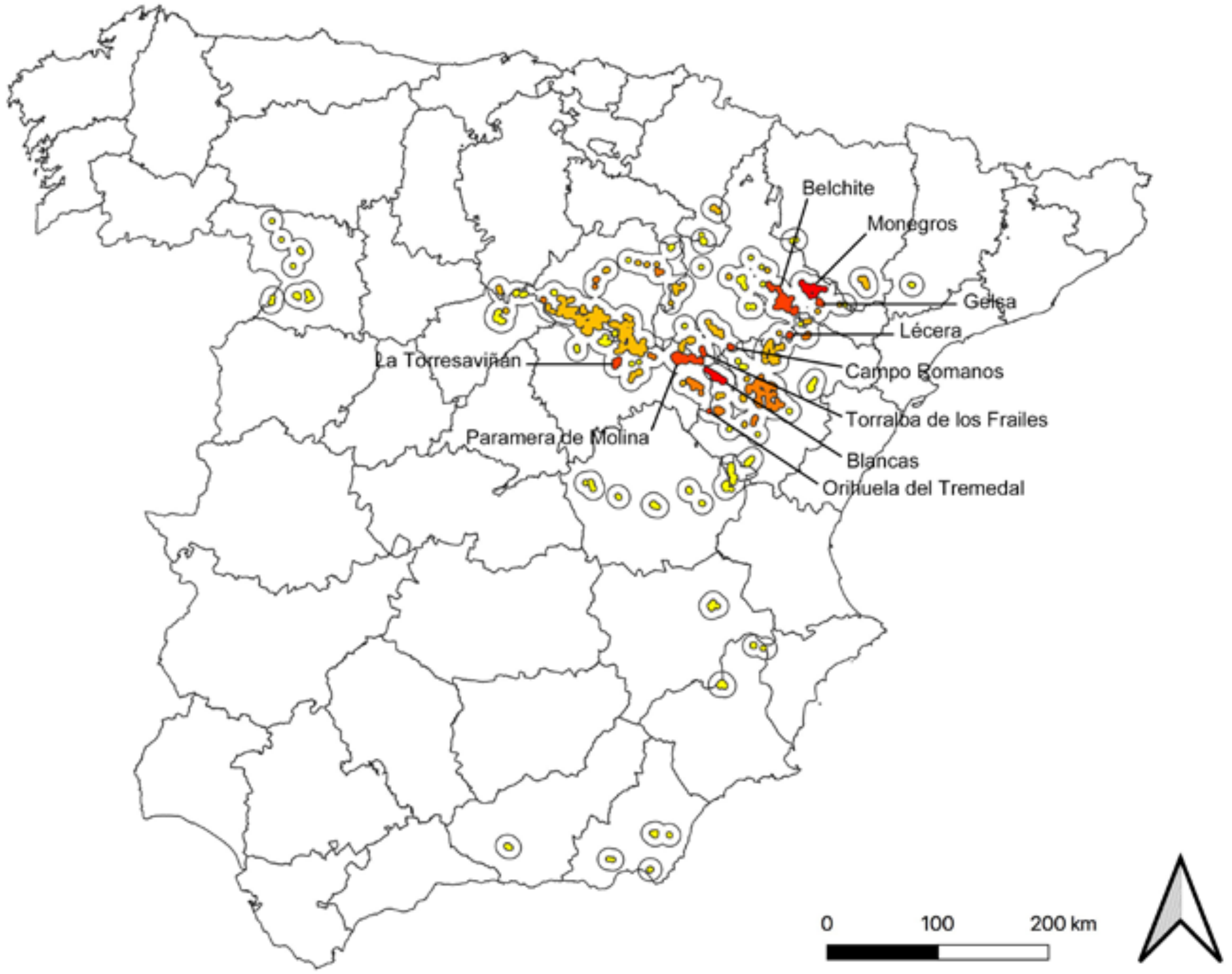

dPCconnector. Contribution of each subpopulation to the connectivity among the rest. 10 most important subpopulations are indicated. Complete list of dPCconnector values in Supplemental Data S3.

Down-left. 5 km movements.

Up-right. 20 km movements.

Down-right. 100 km movements.

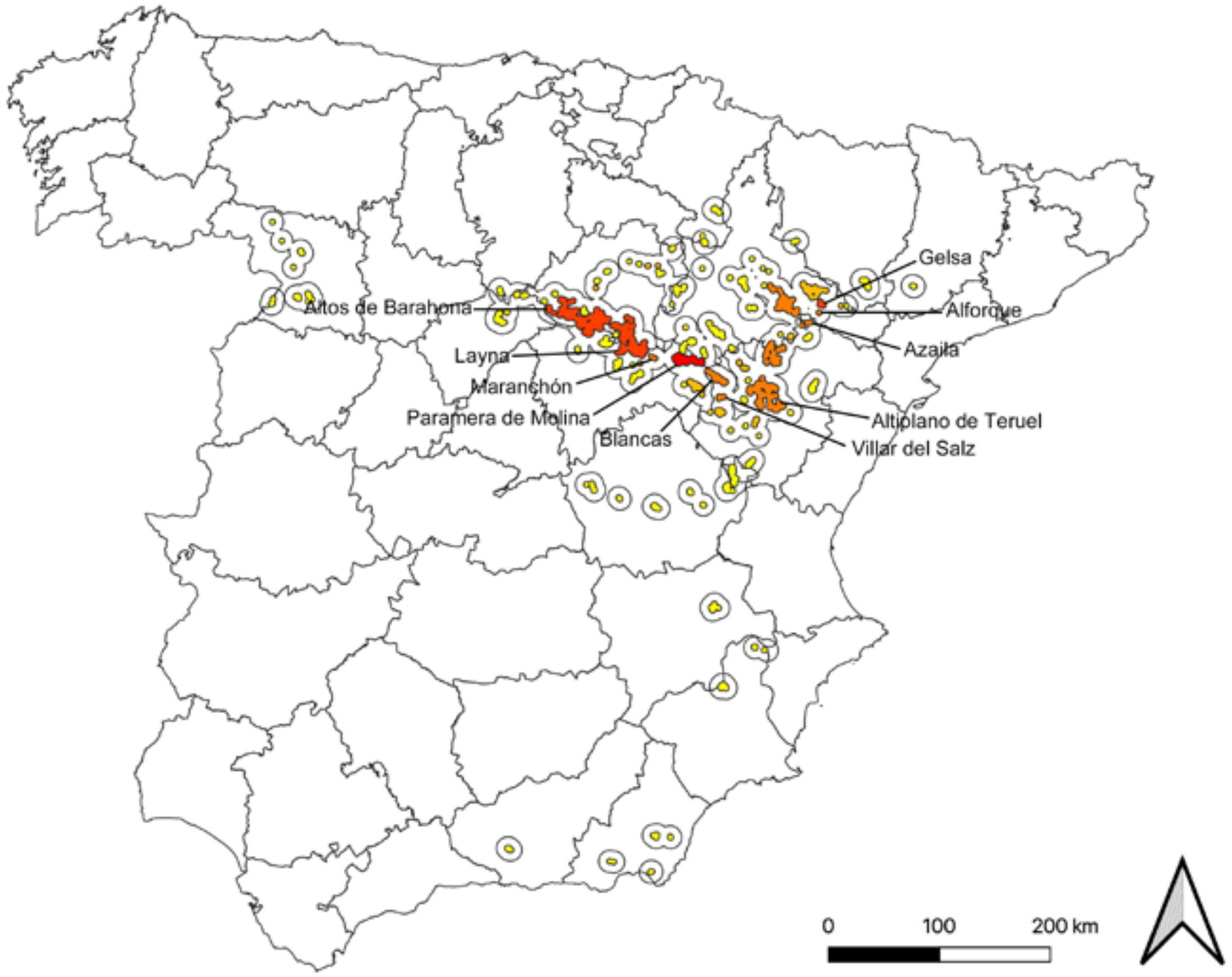

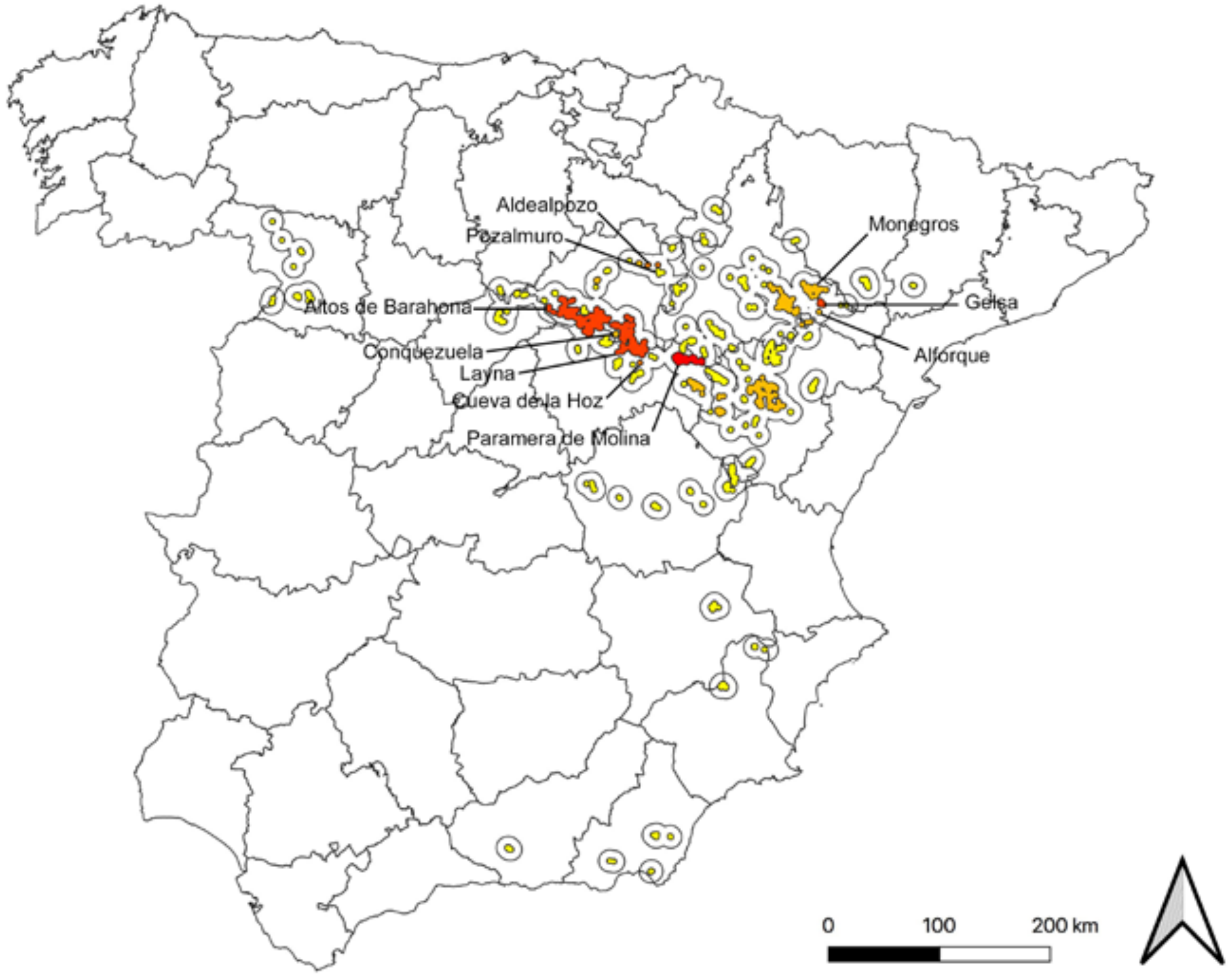

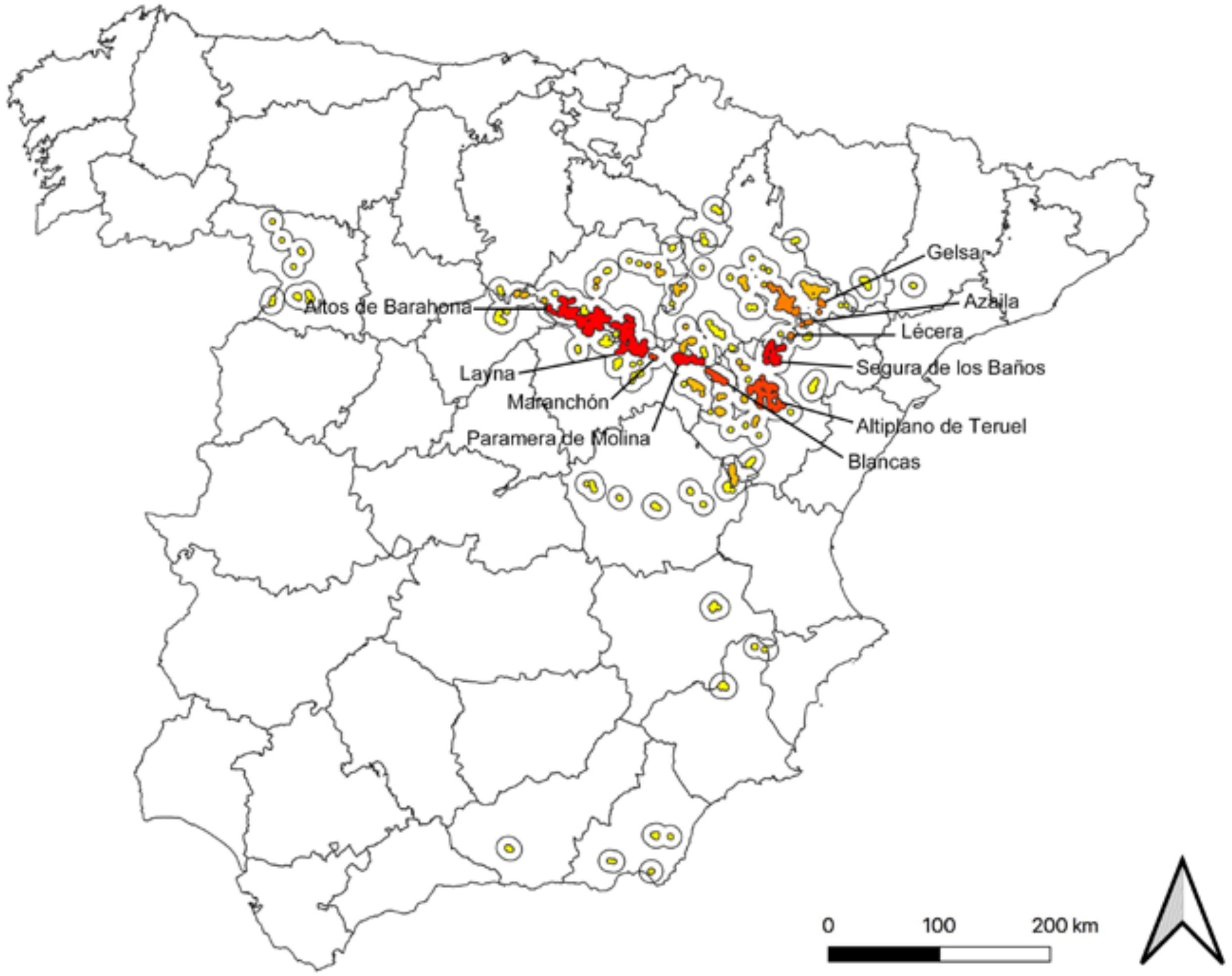

dPC. Global importance of each subpopulation in the connectivity network (sum of dPCintra, dPCflux and dPCconnector). 10 most important subpopulations are indicated. Complete list of dPC values in Supplemental Data S3.

Down-left. 5 km movements.

Up-right. 20 km movements.

Down-right. 100 km movements.

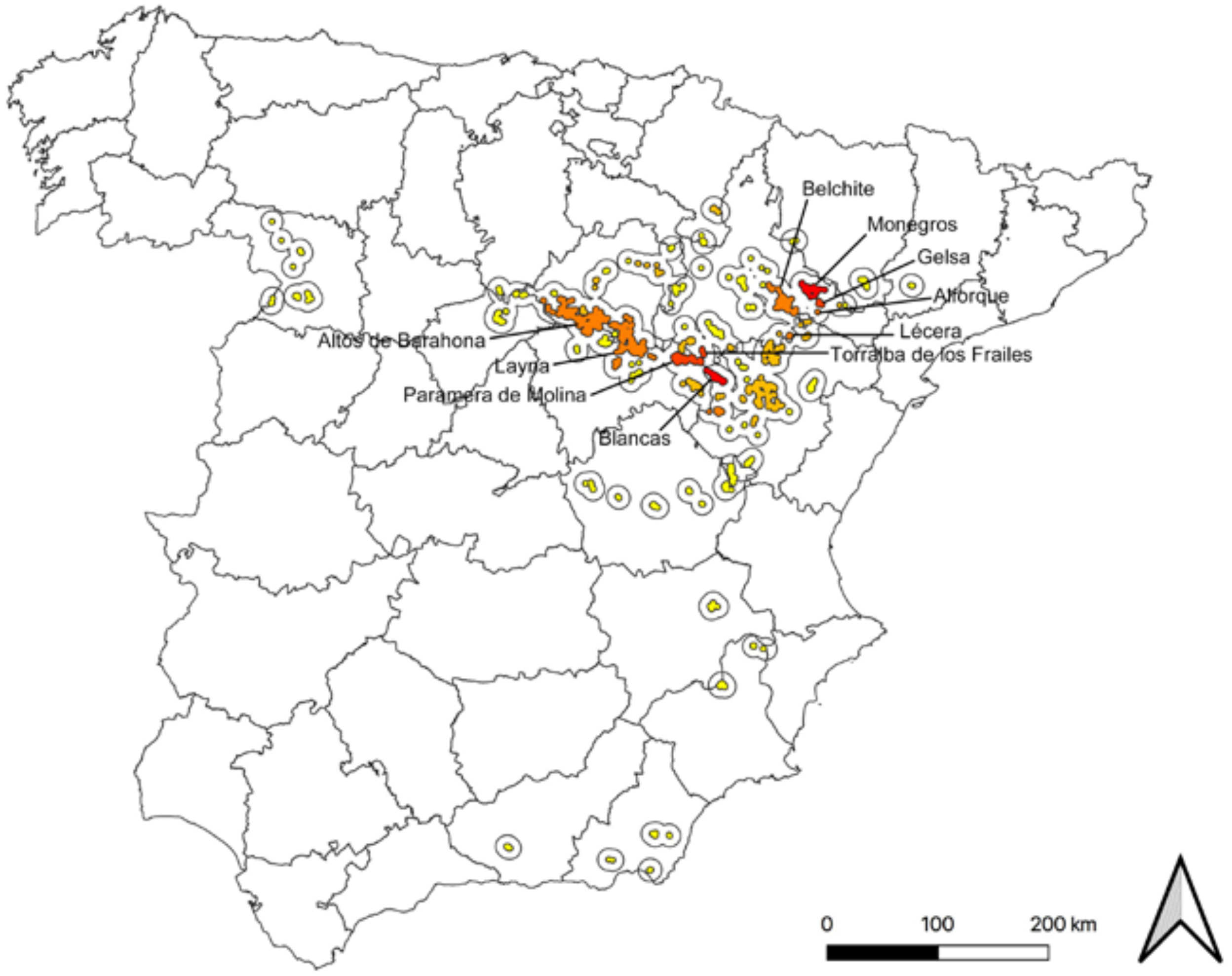

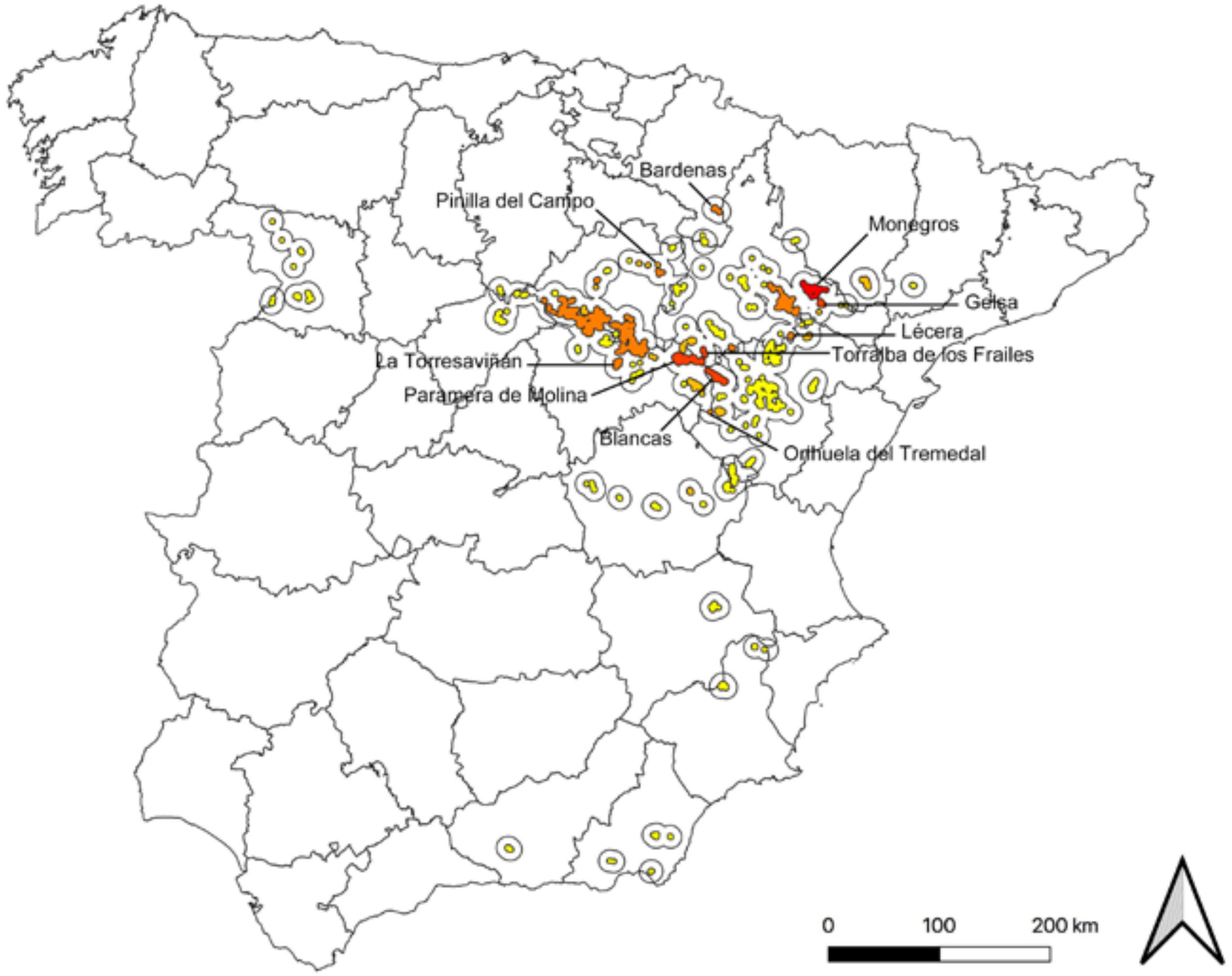

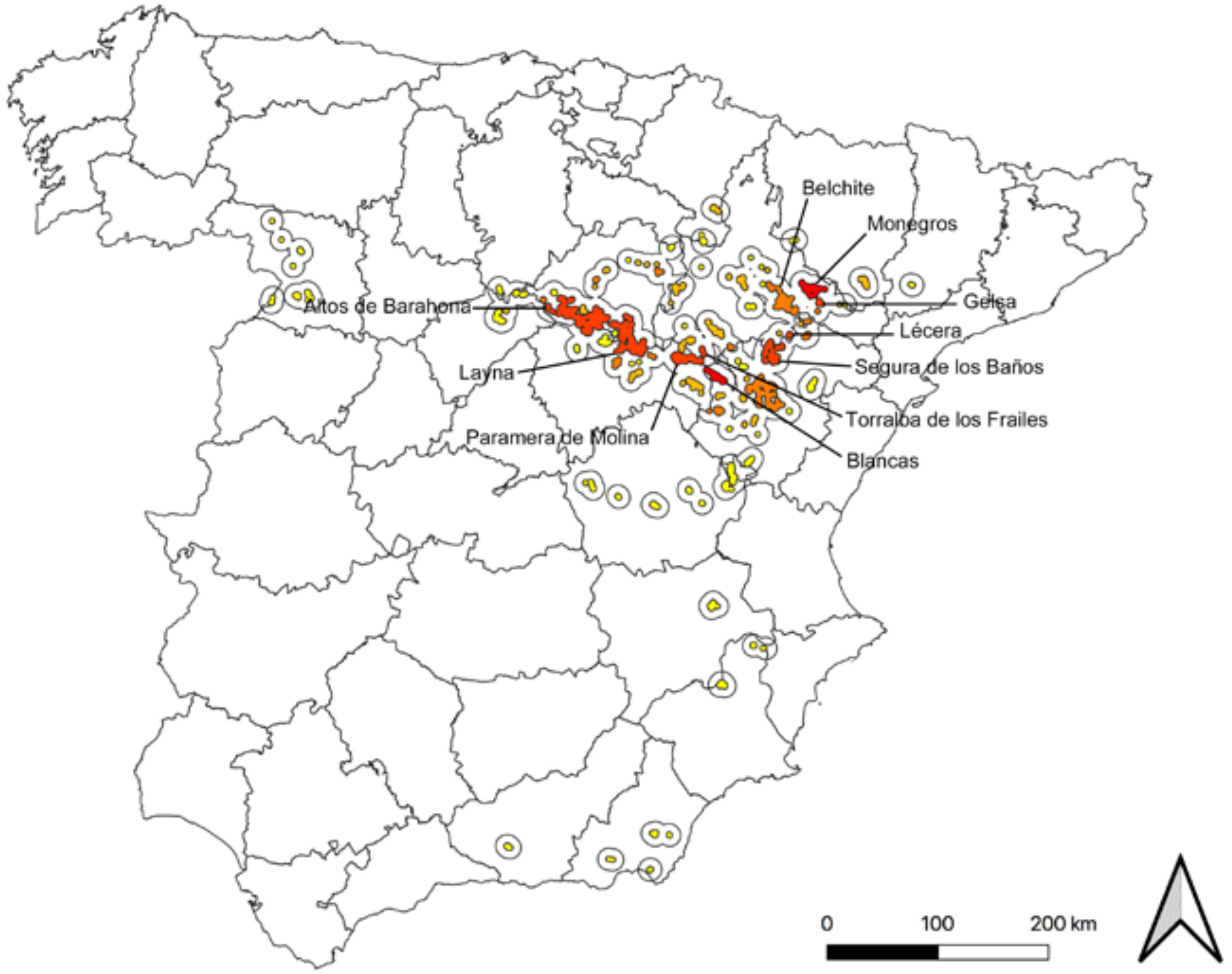

II) PRESENCE OF STEPPING STONES.

dPCintra. Internal quality of subpopulations (intra-patch connectivity), with stepping stones. The 10 most important subpopulations are indicated. Find the complete list of dPCintra values in Supplemental Data S3.

Right figure: dPCintra makes reference to the internal importance of each node and doesn't depend on spatial position. Thus, the ranking is the same for the different movement thresholds and here we only show results of scenario 4 (5 km movements, with stepping stones). Although no stepping stone appears in this top-10 ranking, two of them are positioned in the first 20 values, see the complete list in Supplemental Data S3.

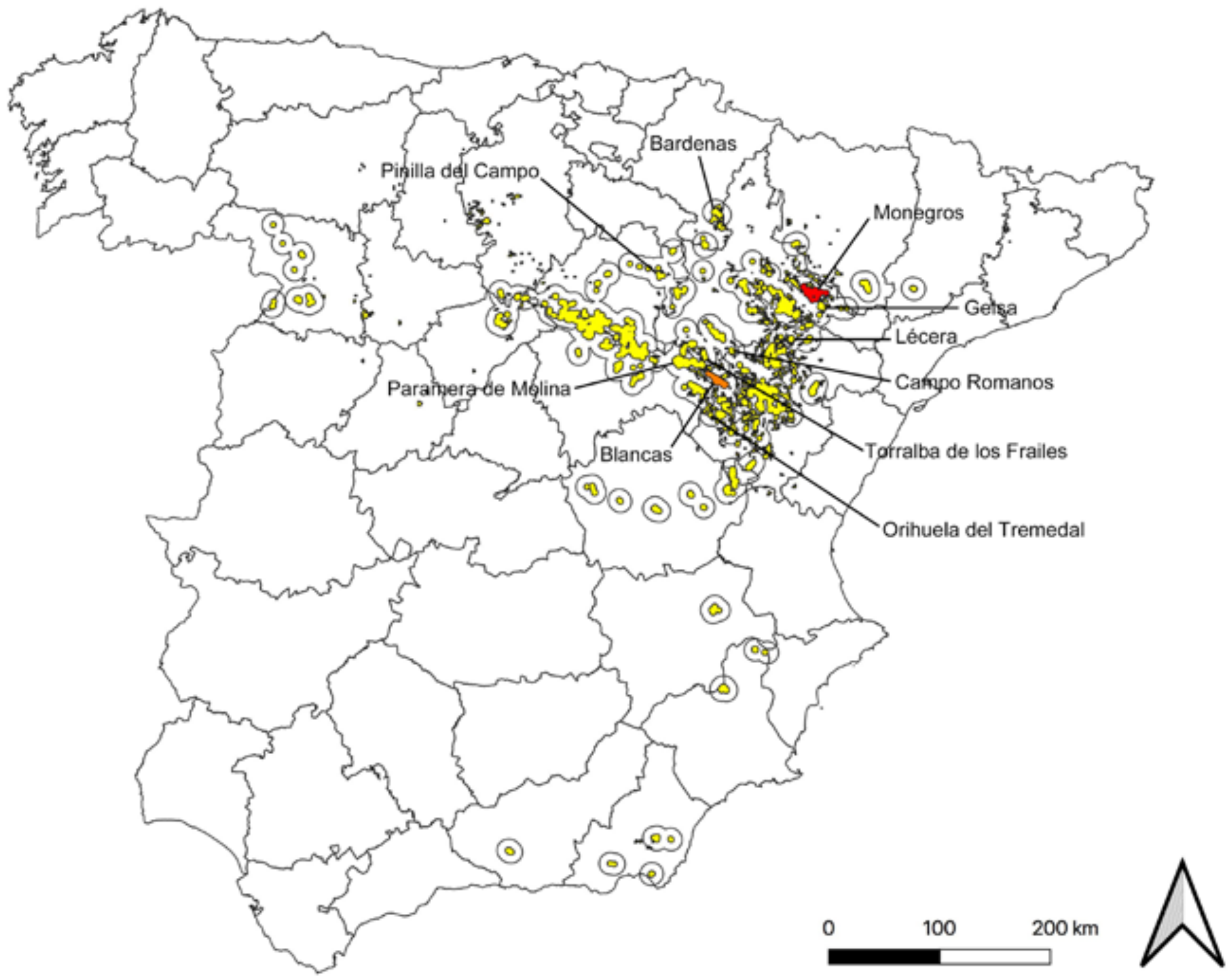

dPCflux. Degree of flow that each subpopulation generates within the network (intra-patch connectivity). The 10 most important subpopulations are indicated. Complete list of dPCflux values in Supplemental Data S3.

Down-left. 5 km movements.

Up-right. 20 km movements.

Down-right. 100 km movements.

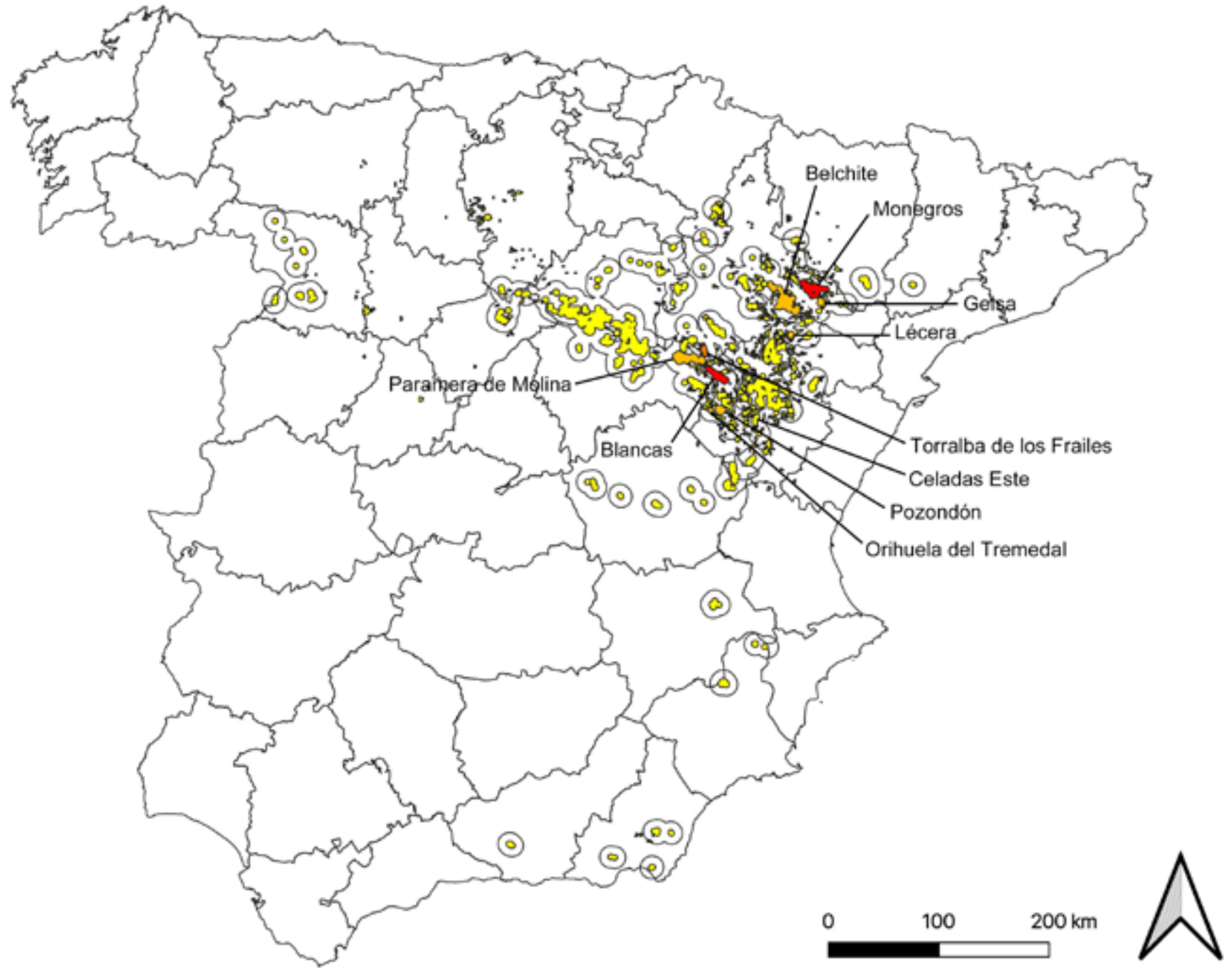

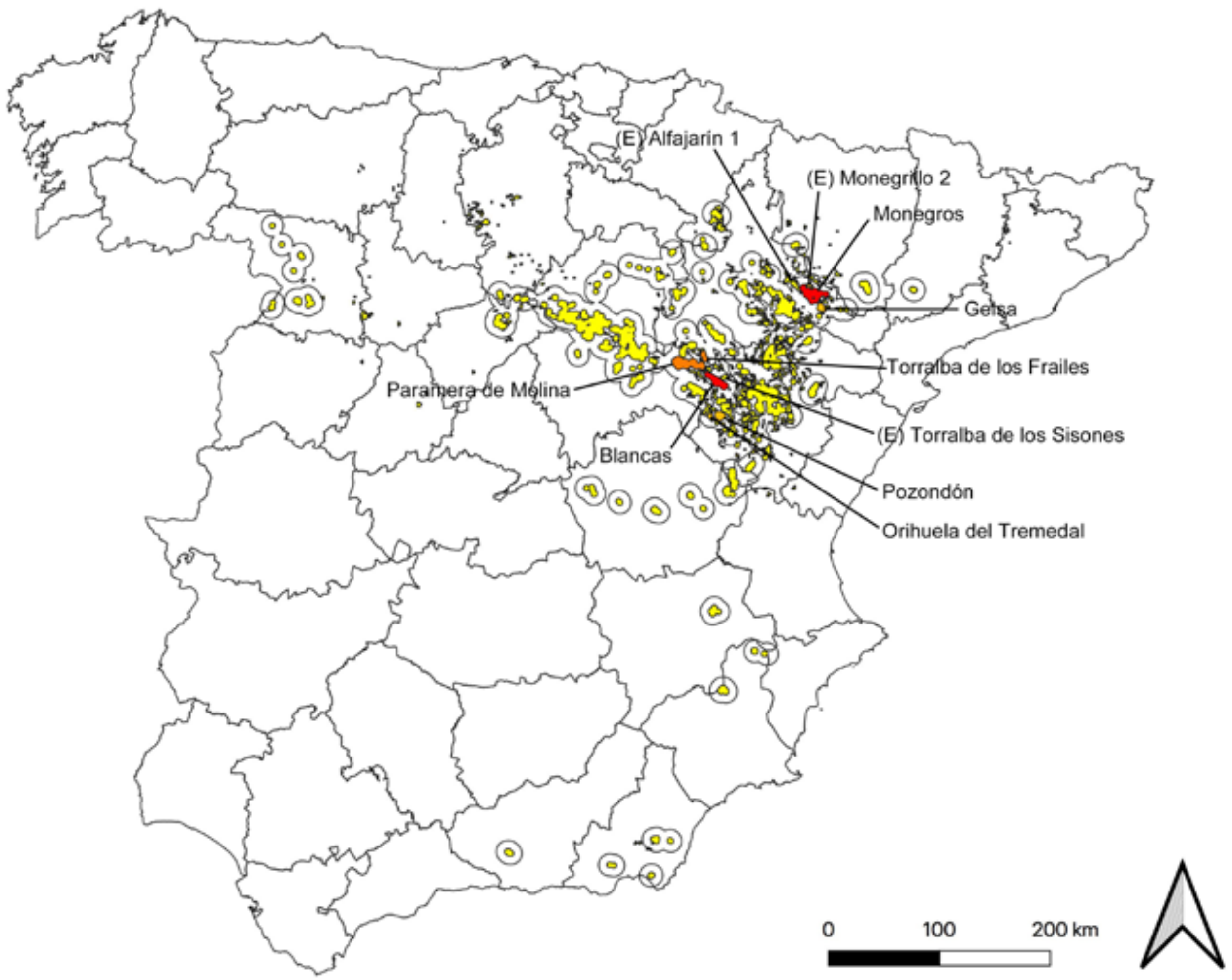

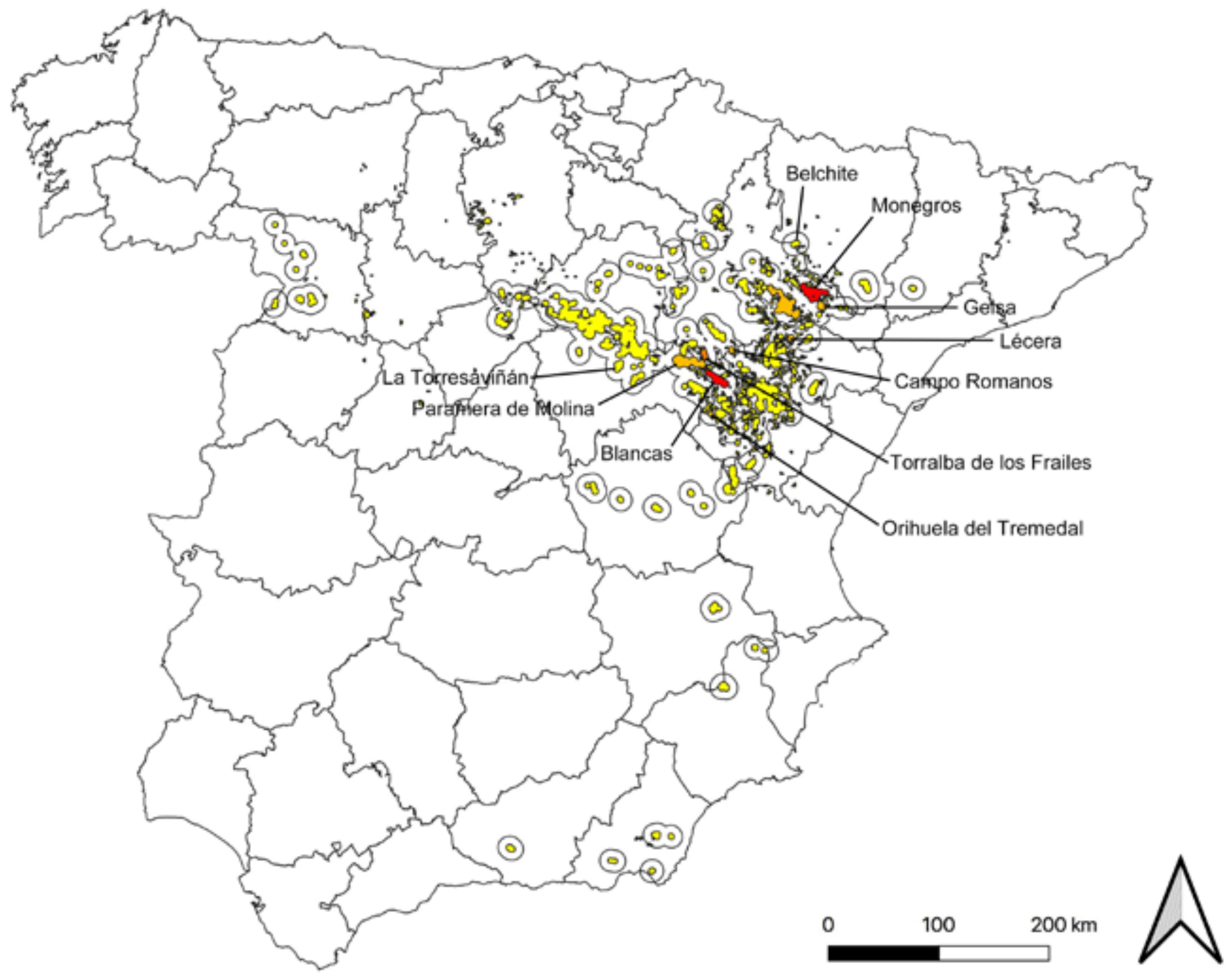

dPCconnector. Contribution of each subpopulation to the connectivity among the rest. 10 most important subpopulations are indicated. Complete list of dPCconnector values in Supplemental Data S3.

Down-left. 5 km movements.

Up-right. 20 km movements.

Down-right. 100 km movements.

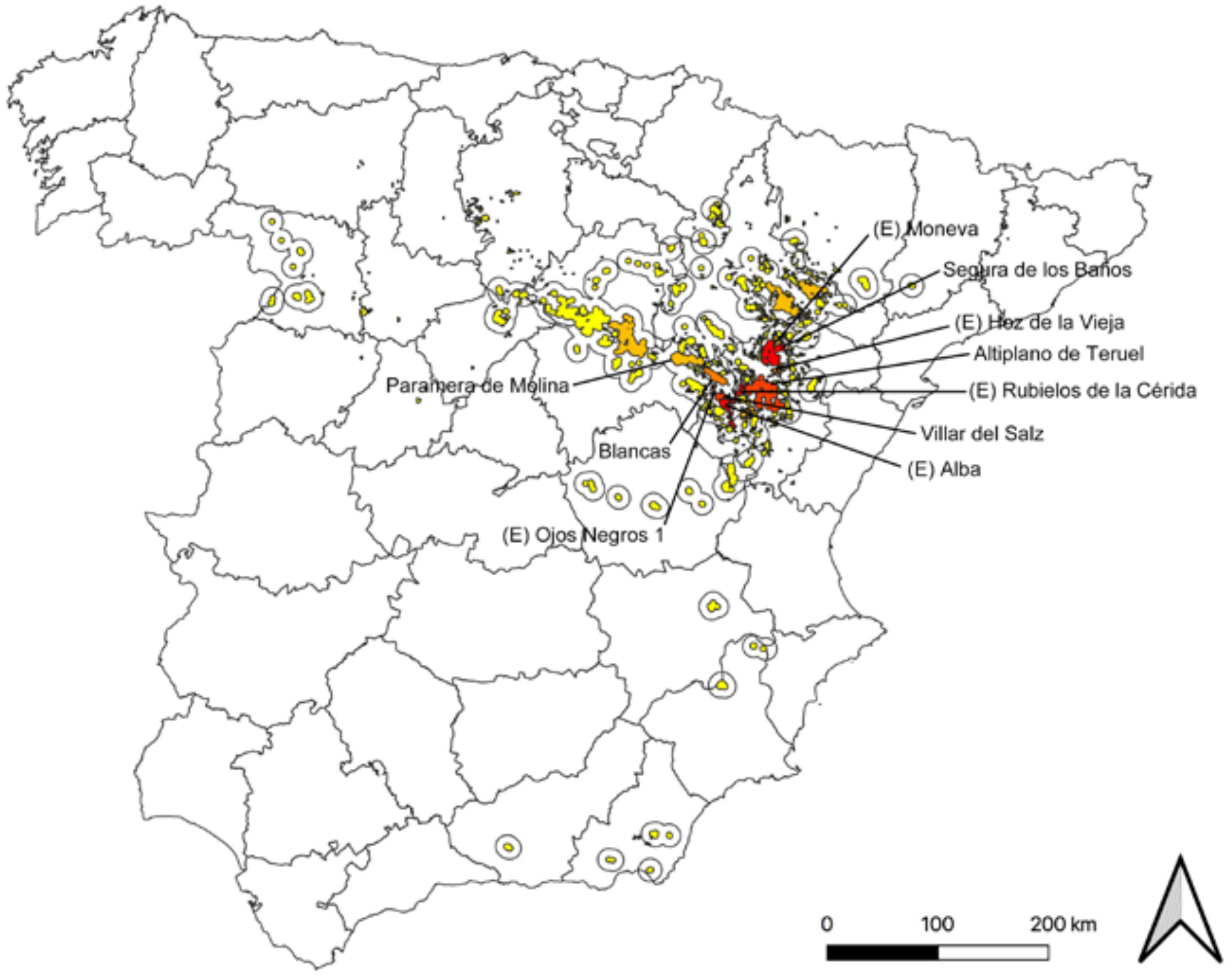

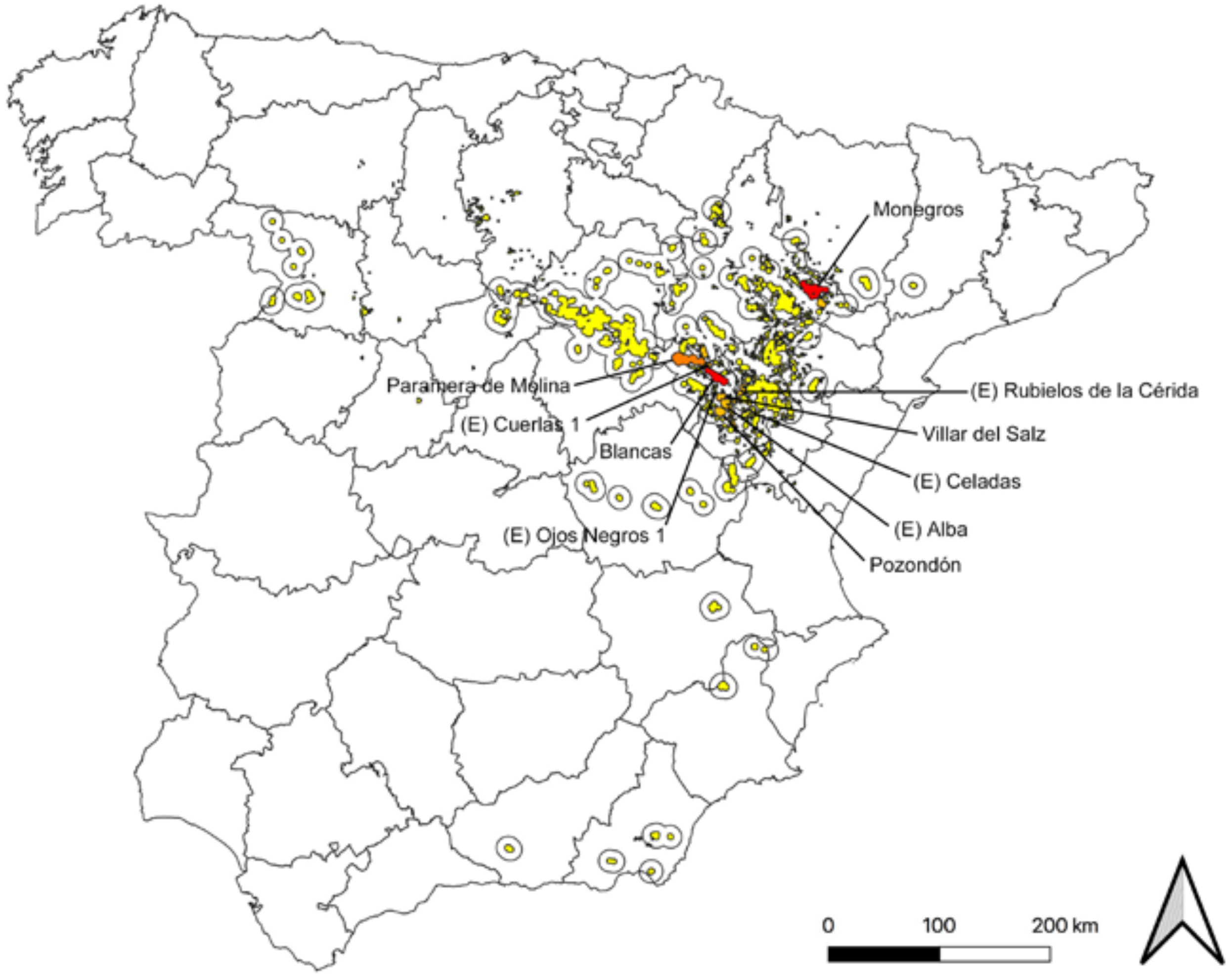

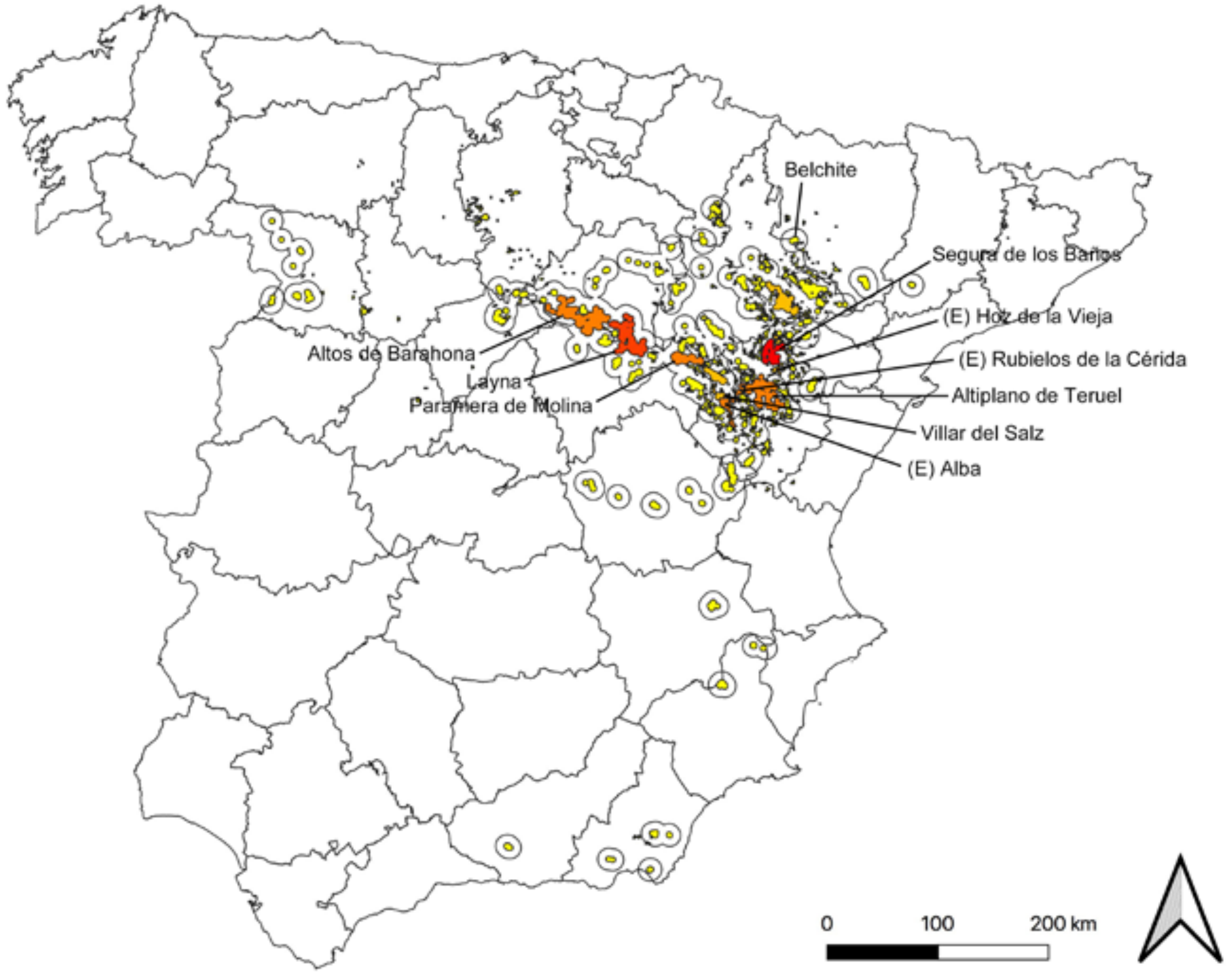

dPC. Global importance of each subpopulation in the connectivity network (sum of dPCintra, dPCflux and dPCconnector). 10 most important subpopulations are indicated. Complete list of dPC values in Supplemental Data S3.

Down-left. 5 km movements.

Up-right. 20 km movements.

Down-right. 100 km movements.

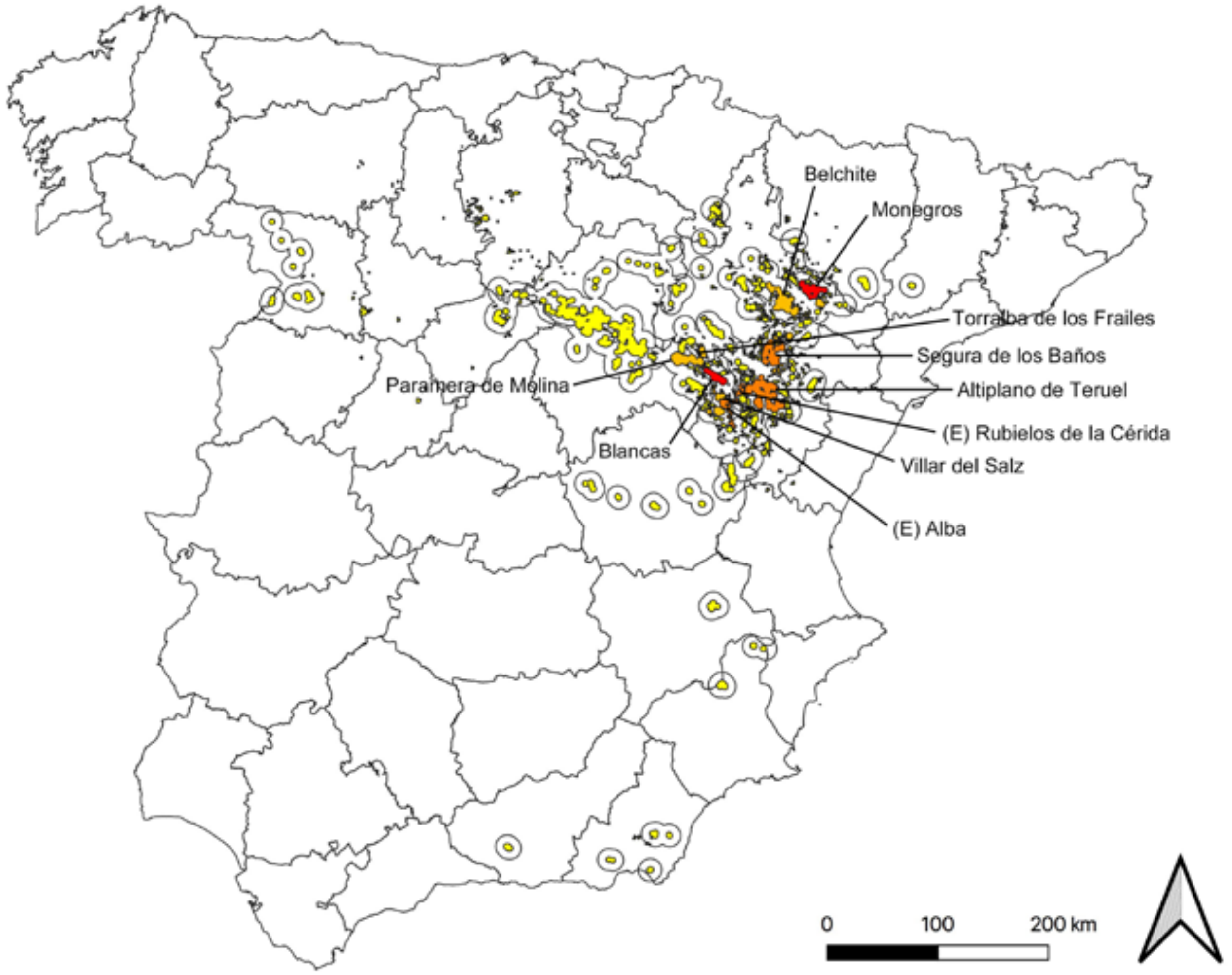

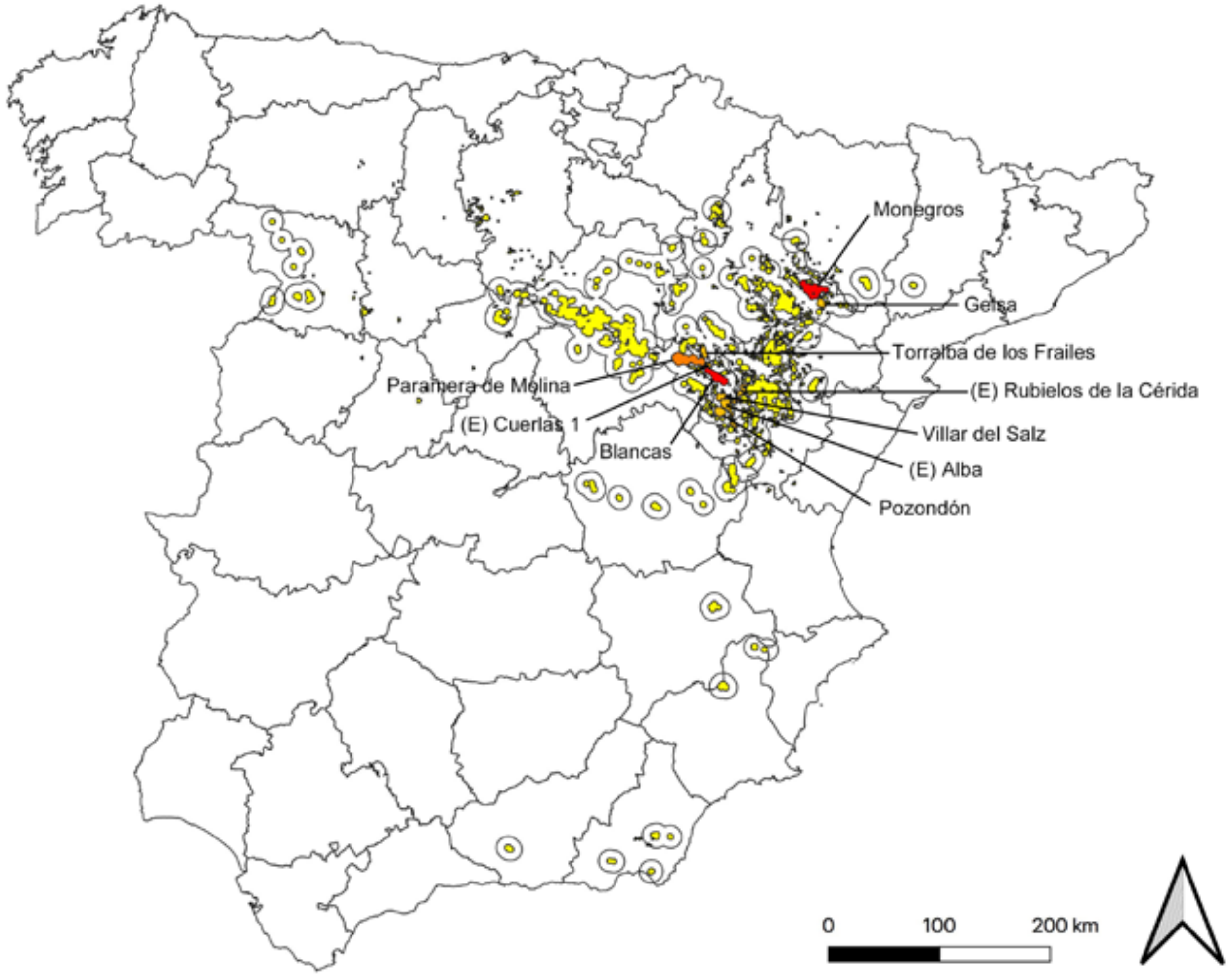

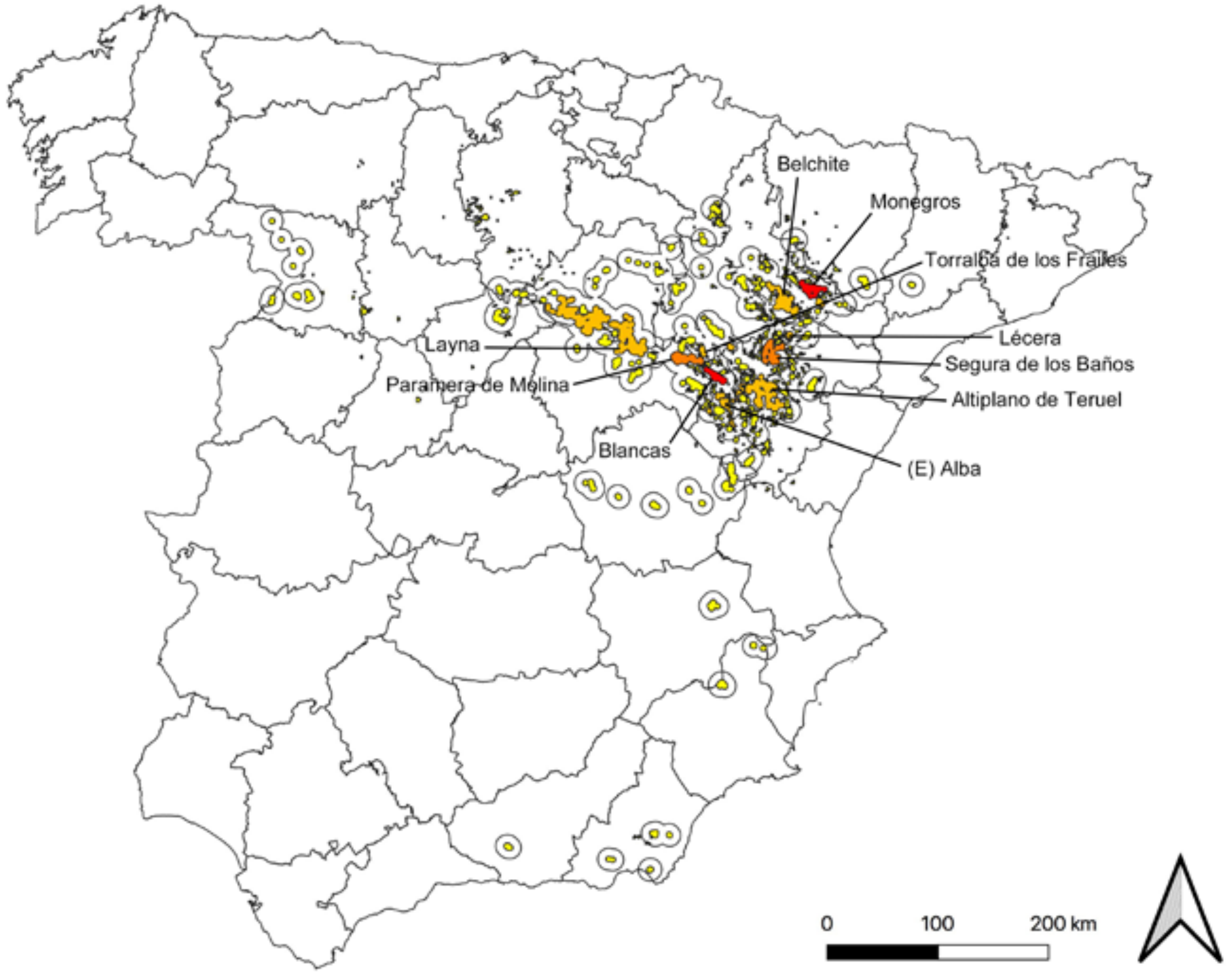

Supplement: Supplemental Information 7 — PDF file with the maps of nodes importance in dPCintra, dPCflux and dPCconnector, under the different scenarios evaluated: movements of 5-20-100 km and presence/absence of stepping stones. [file peerj-09-11925-s007.pdf]
